# Huangqi Guizhi Wuwu decoction alleviates oxaliplatin-induced peripheral neuropathy via the gut-peripheral nerve axis

**DOI:** 10.1186/s13020-023-00826-5

**Published:** 2023-09-07

**Authors:** Zhengwei Zhang, Juan Ye, Xinyu Liu, Wenjing Zhao, Bing Zhao, Xuejiao Gao, Hongli Lan, Yuze Wu, Yang Yang, Peng Cao

**Affiliations:** 1 Jiangsu Provincial Medical Innovation Center, Affiliated Hospital of Integrated Traditional Chinese and Western Medicine, Nanjing University of Chinese Medicine, 100#, Hongshan Road, Nanjing, 210028 China; 2 School of Pharmacy, Nanjing University of Chinese Medicine, Nanjing, 210023 China

**Keywords:** Intestinal flora, Oxaliplatin-induced peripheral neurotoxicity, Huangqi Guizhi Wuwu decoction, Inflammation, Lipopolysaccharide

## Abstract

**Background:**

Oxaliplatin-induced peripheral neurotoxicity (OIPN) limits the dose of chemotherapy and seriously affects the quality of life. Huangqi Guizhi Wuwu Decoction (HGWD) is a classical Traditional Chinese Medicine (TCM) formula for the prevention of OIPN. However, its specific pharmacological mechanism of action remains unknown. Our study found that HGWD can effectively alleviate chronic OIPN and regulate intestinal flora. Therefore, we explored the mechanism of action of HGWD in alleviating chronic OIPN from the perspective of intestinal flora.

**Methods:**

In this study, we established an OIPN model in C57BL/6 mice treated with different concentrations of HGWD. Mechanical pain and cold pain were assessed at certain time points, and samples of mice colon, dorsal root ganglion (DRG), serum, and feces were collected. Associated inflammation levels in the colon and DRG were detected using immunohistochemical techniques; the serum lipopolysaccharide (LPS) levels and associated inflammation were assessed using the appropriate kits; and 16S rRNA sequencing was used to examine the dynamic changes in gut microorganisms. Finally, established fecal microbiota transplantation (FMT) and antibiotic (ABX) pretreatment models were used to validate flora’s role in HGWD for chronic OIPN by pain scoring and related pathological analysis.

**Results:**

HGWD treatment significantly alleviated pain sensitivity in chronic OIPN mice. Pathological results showed that HGWD treatment improved intestinal ZO-1 expression and reduced serum LPS levels and associated inflammatory factors in the colon, serum, and DRG. The 16S rRNA results showed that HGWD restored the composition of the intestinal flora in a time-dependent manner to alleviate OIPN. FMT and ABX experiments demonstrated that HGWD can alleviate chronic OIPN by regulating intestinal flora homeostasis.

**Conclusions:**

HGWD prevents chronic OIPN by dynamically regulating intestinal flora homeostasis, thereby ameliorating intestinal barrier damage and reducing serum LPS and relevant inflammatory factor levels in the colon, serum, and DRG.

**Supplementary Information:**

The online version contains supplementary material available at 10.1186/s13020-023-00826-5.

## Background

The effective survival period of patients with tumors has greatly increased in recent years with advances in tumor diagnosis and treatment methods [1]. However, chemotherapy-induced peripheral neuropathy (CIPN) is a significant challenge for patients with clinical tumors [2, 3]. CIPN manifests as sensory, motor, or autonomic deficits of varying severity with symptoms such as numbness, tingling, altered tactile sensation, sensory abnormalities, and cold hypersensitivity that progress to sensory loss in severe cases [4]. Platinum drugs are the most neurotoxic, with the highest incidence of oxaliplatin-induced peripheral neurotoxicity (OIPN), which induces two main types of peripheral neurotoxic reactions in clinical treatment [5]. The first type is acute peripheral neurotoxicity with a prevalence of 85–96%, which occurs within a few hours of treatment with oxaliplatin injection, is short-lived, and resolves spontaneously when the drug is discontinued [6, 7]. The second type is chronic peripheral neurotoxicity, which occurs clinically when patients receive continuous doses above 780–850 mg/m^2^ and has a prevalence of 68–98% [8]. Once OIPN occurs, recovery is extremely difficult, and patients have to receive reduced chemotherapy dose or even stop taking it, which greatly reduces the effect of tumor prevention and the quality of life [9]. No drug or treatment regimen has a definitive curative effect, especially in chronic OIPN [3]. Therefore, research on their pathogenesis and prevention strategies is of great scientific and societal importance.

The intestinal flora can regulate pain and is involved in the development of CIPN [10–13]. In an experiment with sterile mice and ABX-pretreated mice, it was found that temporary eradication of gut microbiota prevents oxaliplatin-induced mechanical hyperalgesia, and its protective effect disappeared after restoring the intestinal flora [14]. The translocation of gut microbiota or their metabolites, such as lipopolysaccharide (LPS), into the circulation can induce chronic inflammation [15]. The imbalance of gut microbiota and its metabolites will increase the excitability of nociceptors by activating inflammatory cells and glial cells to promote the release of related inflammatory factors (such as IL-6 and TNF-α) [11, 16–18]. In addition, there is a close association between the intestinal microbiota and the mucosal layer of the intestine, and an increase in harmful microbiota affects intestinal tight junctions and alters intestinal permeability [19–21]. LPS enters the blood, promotes the production of multiple pro-inflammatory factors, and triggers chronic systemic inflammation when intestinal permeability is altered [22]. High mobility group box 1 (HMGB1) is a pathogenic inflammatory factor that readily binds to other pro-inflammatory molecules, synergistically triggers an inflammatory response, and is a poor factor in promoting neuroinflammation and pain development [23–25].

TCM has shown promising results in preventing OIPN because of its synergistic effects with multiple targets and levels [26]. HGWD is a classic recipe in the Synopsis of the Golden Chamber [27]. HGWD significantly reduced the incidence of clinical OIPN patients [28]. Related animal studies have also found that HGWD protects against chemotherapy-induced neurotoxicity by inhibiting the inflammatory response and activating antioxidant functions [29]. We found that HGWD had good therapeutic effects on chronic OIPN. However, its specific pharmacological mechanism of action remains unknown and requires further investigation.

In recent years, TCM and gut microbiota have been the research focus [30–32]. TCM plays a key role in maintaining gut microbiota homeostasis in treating diseases [33]. However, the role of gut microbiota in HGWD for chronic OIPN is unclear. Therefore, here, we designed an experiment using an OIPN mouse model treated with different concentrations of HGWD, dynamically detected intestinal barrier function, serum LPS content, and inflammatory expression levels in the colon, DRG, and serum of mice samples, and dynamically analyzed the changes in intestinal flora in OIPN mice treated with HGWD at different periods by 16S rRNA sequencing. Finally, fecal microbiota transplantation (FMT) and antibiotic (ABX) experiments were performed to validate flora’s role in HGWD to alleviate chronic OIPN. This is the first study to demonstrate a good therapeutic effect of HGWD in chronic OIPN. Our findings may provide a basis for the regulation of microbial homeostasis to prevent OIPN development.

## Methods

### Antibodies and drugs

Oxaliplatin (T0164), Neomycin (T0950), Metronidazole (T1079), Vancomycin (T0832), and Ampicillin (T6386) were purchased from TargetMol, Boston, USA. HMGB1 (ab79823), ZO-1 (ab221547), IL-6 (ab208113), TNF-α (ab1793) were purchased from abcam, USA. HGWD consists of *Astragalus mongholicus* Bunge (Huangqi) 18 g, *Neolitsea cassia* (L.) Kosterm (Guizhi) 9 g, *Paeonia lactiflora* Pall (Baishao) 9 g, *Zingiber officinale* Roscoe (Shengjiang) 9 g, *Ziziphus jujuba* Mill (Dazao) 9 g, as shown in Table 1. All plant names have been checked by MPNS (http://mpns.kew.org). All the herbs were placed in a round bottom flask, add 10 times ultrapure water and soak for 1 h, and heated and decocted for 1 h. The liquid was filtered out through gauze. Subsequently, 5 times ultrapure water was added to the residue, it was boiled for 30–60 min, and the liquid was filtered out. Finally, the two solutions were mixed and concentrated to make 2 g/mL of aqueous extract concentrate. HGWD was provided by the pharmacy of Jiangsu Provincial Hospital of Integrative Medicine, with clear open source, according to the Chinese Pharmacopoeia (2015 version) [29]. Our group pre-screened the active compounds of HGWD and performed relevant quantitative and qualitative analyses of HGWD, the concentrations of the main components were determined by external standard curves (Additional file 2) [28, 29].

**Table 1:** The compositions of HGWD.

Herbal name	Accepted scientific name	Dose (g)
Huangqi	*Astragalus mongholicus* Bunge	18
Guizhi	*Neolitsea cassia* (L.) Kosterm	9
Baishao	*Paeonia lactiflora* Pall	9
Shengjiang	*Zingiber officinale* Roscoe	9
Dazao	*Ziziphus jujuba* Mill	9

### Animal

**Fig. 1:**
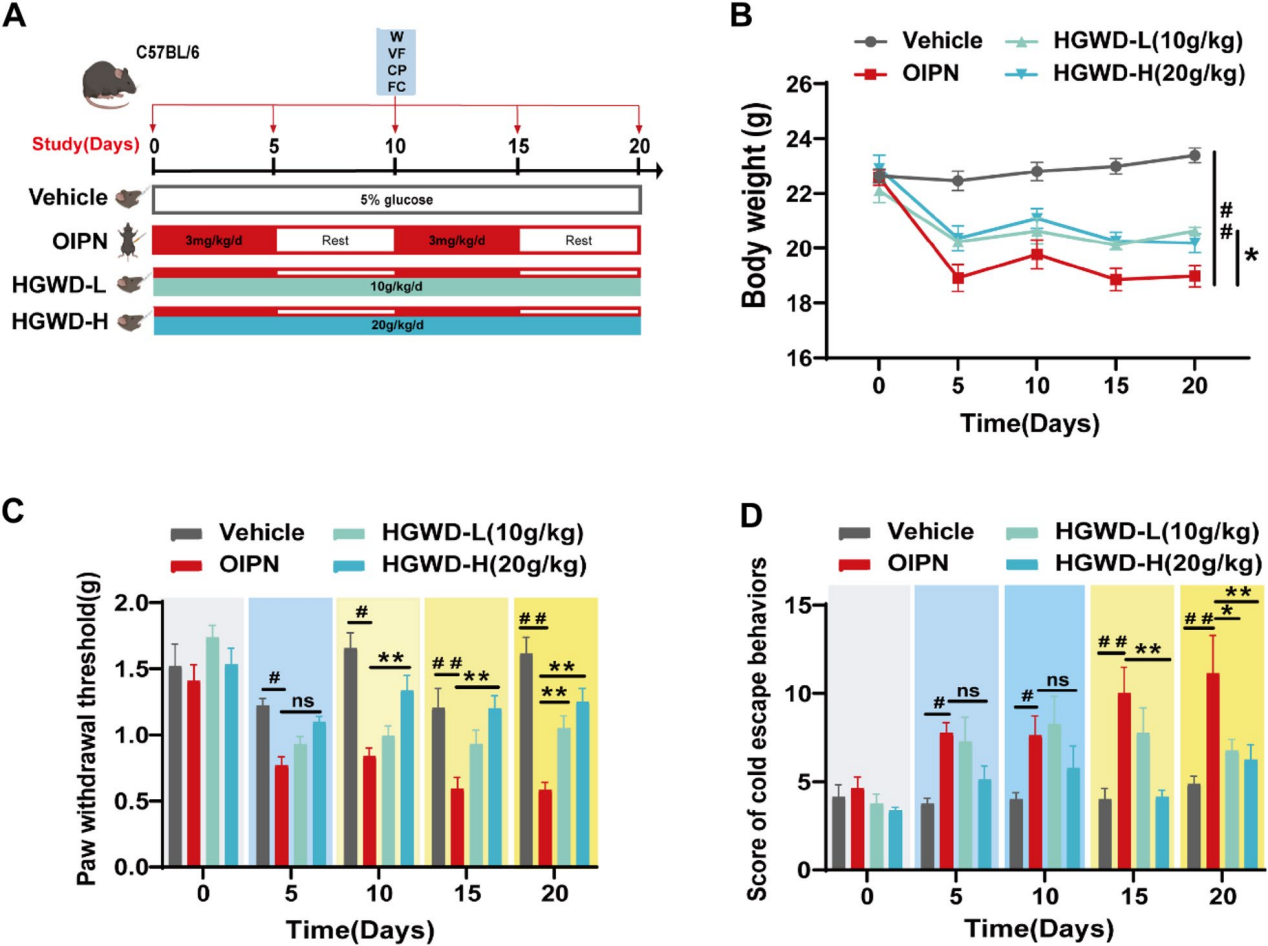
Therapeutic effect of HGWD on OIPN. **A** HGWD treatment of OIPN animal model. **B** Body weight test of mice. **C** Paw withdrawal threshold induced by mechanical stimulation (Von Frey filament). **D** Escape behavior score by cold stimulation (4 ℃). Data are expressed as mean ± SEM. HGWD-L: 10 g/kg, HGWD-H: 20 g/kg. #P < 0.05, ##P < 0.01 (compared with Vehicle group); *P < 0.05, **P < 0.01 (compared with OIPN group)

Male C57BL/6 mice (6–8 weeks, 20–22 g) were purchased from Jiangsu Huachuang Xinnuo Pharmaceutical Technology Co, China (License No.: SCXK(SU)2020-0009). All mice were housed in a standard experimental environment (temperature 22 ± 2 °C, humidity 50 ± 10%, 12-h light/dark cycle) and given food and water. Jiangsu Institute of Traditional Chinese Medicine Animal Care and Use Committee approved all procedures involving animals (Approval No.: SYXK(SU)2021-0025), and all details of the procedures were written according to the ARRIVE guidelines [34]. After 2 weeks of habituation in a fixed environment and measurement of the pain-sensitive baseline, four groups were randomized: Vehicle group, OIPN group, low dose HGWD group (10 g/kg), and high dose HGWD group (20 g/kg). We measured mechanical pain and cold pain on days 0, 5, 10, 15, and 20, and colon, serum, DRG, and fecal samples were collected. All behavioral assessments were performed by the same person in a double-blind fashion. The specific design is shown in Fig. 1A.

### FMT experiments

We designed FMT experiments according to previous literature reports [35, 36]. After 2 weeks of adaptation to the standard experimental environment, six groups were randomized: Vehicle group, Vehicle + HGWD group, OIPN group, OIPN + HGWD group, OIPN + FMT-VH (collection of feces from Vehicle + HGWD group mice for FMT) group, OIPN + FMT-OH (collection of feces from OIPN + HGWD group mice for FMT) group. Vehicle group: The mice in this group received an intraperitoneal injection of 100 µL 5% glucose injection (oxaliplatin was dissolved in 5% glucose injection to ensure consistent experimental conditions and to exclude the effect of 5% glucose injection on the intestinal flora of mice; the Vehicle group received 5% glucose solution as solvent control). OIPN group: intraperitoneal injection of 100 µL oxaliplatin reagent is continuously administered for two cycles (5 days of dosing and 5 days of rest for one cycle). Vehicle + HGWD group: 200 µL HGWD (20 g/kg) by gavage daily for treatment and then intraperitoneal injection of 100 µL 5% glucose 2 h later; OIPN + HGWD group: 200 µL HGWD (20 g/kg) by gavage daily for treatment and then intraperitoneal injection of 100 µL oxaliplatin reagent 2 h later (Vehicle + HGWD group and OIPN + HGWD group provide the source of fecal bacteria for the treatment group). First, fresh feces from mice in the Vehicle + HGWD and OIPN + HGWD groups were collected once every other day and pooled from the same group. Then, 100 mg of fresh stool was resuspended in 1 mL of sterile water, and a desktop vortex machine was used to mix it vigorously, making the feces completely suspended in sterile water. Finally, it was centrifuged at 800 ×*g* speed for 3 min, and the suspension was collected. The suspension was centrifuged at 3099×*g* speed for 10 min, and the deposited bacteria were collected and suspended in sterile water for FMT. The OIPN + FMT-VH and OIPN + FMT-OH groups should transplant the suspension of the feces of the donor group into the corresponding recipient group mice 2 h in advance and then inject oxaliplatin intraperitoneally. FMT gavage treatment was performed once every other day, with 200 µL of fecal suspension gavage into each mouse each time. The specific experimental design is shown in Fig. 4A.

### ABX pre-processing experiments

All mice were acclimated for 2 weeks, pseudo-sterile mice were induced by providing ABX drinking water (ABX: Neomycin 0.5 g/L, Metronidazole 0.5 g/L, Vancomycin 0.25 g/L, and Ampicillin 0.5 g/L) in advance for 14 days [37, 38], and the mice were categorized into three groups in the presence of ABX drinking water at all times: Vehicle + ABX group, OIPN + ABX group, OIPN + ABX + HGWD group. Vehicle + ABX group: intraperitoneal injection of 100 µL 5% glucose injection, OIPN + ABX group: intraperitoneal injection of 100 µL oxaliplatin reagent is continuously administered for two cycles. OIPN + ABX + HGWD group: 200 µL HGWD (20 g/kg) by gavage daily for treatment and then intraperitoneal injection of 100 µL oxaliplatin reagent 2 h later. The specific experimental design is shown in Fig. 6A.

### Measurement of mechanical pain and cold pain

The mechanical pain threshold and total cold hyperalgesia score in mice’s left/right lower limbs were measured using a double-blind method on days 0, 5, 10, 15, and 20. For the measurement method of mechanical pain and the detailed scoring rules of cold pain, please refer to the previous experimental design of the research group [39].

### Mechanical pain

Mice were first placed in a mesh-bottomed Plexiglas cage and allowed to acclimate for 5–10 min. Mice were precisely stimulated with Von Frey fiber filaments in the middle of the left/right hind paw, avoiding the insensitive thick paw pad area. The stimulation intensity started at 0.6 g of fiber filament, which caused the fiber filament to bend for a few seconds, and the foot-shrinking response of the experimental mice was observed; if the mice showed a foot-shrinking response, it was marked as a positive response. If there were three positive responses in five consecutive stimuli, they were marked as X; otherwise, they were marked as O. A response was reduced to the previous pressure level, and a lack of response was increased to the latter level. Repeated for six rounds. Finally, statistical analysis of the final data.

### Cold pain

Mice were acclimatized for 30 min before cold pain was measured. The temperature was maintained at 4 °C during the measurement, and the total measurement time was 60 s. The escape behavior of the mice was recorded and scored during the measurement time (0 = no response; 1 = mild cold escape response, such as lifting the hind foot or walking backwards; 2 = strong cold escape response, such as jumping up), and the sum of the scores during the measurement time was counted.

### Measurement of inflammatory factors and LPS

ELISA reagent kit was used and relevant inflammatory factors (IL-6, TNF-α, HMGB1) in serum were detected according to the strict instructions of the kit. Serum LPS levels were determined by the Limulus test. The mouse IL-6 ValukineTM ELISA kit (VAL604) was purchased from Novus Biologicals, USA. Mouse TNF-α ELISA KIT (E-MSEL-M0002), Mouse HMGB1 ELISA KIT (E-EL-M0676c) was purchased from Elabscience, USA. Endotoxin assay TAL kit (EC80545S) was purchased from Xiamen Baiande, China.

### Immunohistochemical analysis

Expression of ZO-1, IL-6, TNF-α and HMGB1 in colon and DRG samples was evaluated by immunohistochemistry (IHC). Fixed sample from the mouse colon and DRG in 4% paraformaldehyde and the samples were processed by paraffin embedding, sectioning, dewaxing, hydration, removal of endogenous catalase, antigen retrieval, blocking, antibody incubation, and staining. Finally observed and photographed. Using Image Pro Plus 6 software, we assessed immune responses and calculated the area density of the specimens.

### 16S rRNA gene sequencing and data analysis

We collected fresh feces from mice at a specified time and frozen them at − 80 °C. The E.Z.N.A^®^ Soil DNA Kit (Omega Bio-Tech, Norcross, Georgia, USA) was used to extract microbial DNA from fecal. The V4-V5 region of the 16S ribosomal RNA gene of bacteria was amplified by PCR using primers 515 F 5′-barcode-GTGGCCAGCMGCCCGG-3′ and 907 R 5′-CCGTTCAATTCMTTTRAGTTT-3′ (95 °C 20 s, 55 °C 30 s, 72 °C 30 s, and extended for 5 min). Using the AxyPrep DNA Gel Extraction Kit (Axygen Science, Union City, California, USA) amplicons were extracted from 2% agarose gels and purified. Qubit^®^ 3.0 Quantitative was used to quantify the purified PCR product, Illumina platform (Shanghai BIOZERON Biotechnology Co., Ltd.) (2 × 250) was used to sequence pairs of ends. A similarity of 97% was obtained with UPARSE (version 7.1). In addition, principal component analysis (PCA) was conducted using the Ayanami microclass platform.

### Statistical analysis

We present all experimental data as mean ± SEM. One-way ANOVA and Student’s t-test were used to confirm the significance between the groups. Unless otherwise stated, all experimental data were plotted using GraphPad Prism 9.0. P < 0.05 was regarded as statistically significant.

## Results

### HGWD effectively alleviates chronic pain response in OIPN mice model

We constructed an OIPN mouse treatment model via intraperitoneal injection of 3 mg/kg oxaliplatin for two cycles (5 days of dosing and 5 days of rest for one cycle) and gavage of low-dose (10 g/kg) and high-dose (20 g/kg) HGWD (Fig. 1A). During the induction of the OIPN mice model, oxaliplatin administration significantly reduced the body weight of the mice, most significantly after 5 days of administration (Fig. 1B). Compared to that in the Vehicle group, the continuous accumulation of oxaliplatin in mice significantly reduced the mechanical pain threshold (Fig. 1C) and increased the cold pain escape behavior score (Fig. 1D). Next, treatment with HGWD improved the body weight of OIPN mice, and with an increase in the treatment time, both high-dose and low-dose administration groups showed a significantly reduced high cold pain escape score following induction by oxaliplatin and an increase in mechanical pain threshold, which was somewhat dose-dependent. Moreover, we found that the improvement effect of HGWD on mechanical and cold pain in OIPN mice was more obvious in the later stages of the model (Fig. 1C, D). The experiment shows that HGWD significantly improved chronic pain in OIPN mice.

### HGWD alleviation of chronic OIPN is related to regulating intestinal, serum, and DRG inflammatory infiltration

**Fig. 2:**
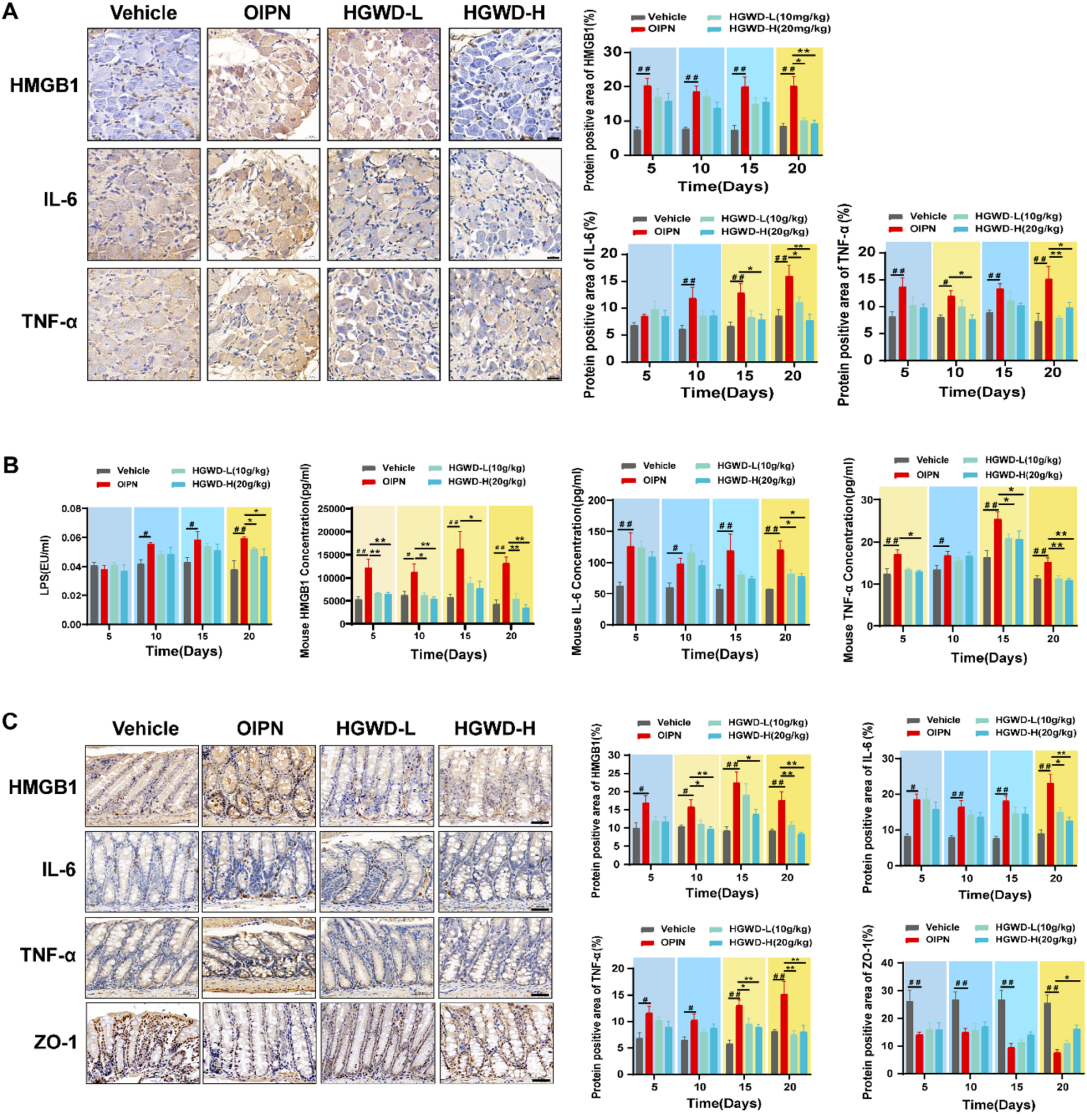
Effect of HGWD treatment on intestinal barrier, serum LPS, and DRG, serum, colon inflammation in OIPN mice. **A** The expression level of HMGB1, IL-6, and TNF-α in the DRG of OIPN mice treated with HGWD (magnification: 500×). **B** Relative levels of HMGB1, IL-6, TNF-α, and LPS in the serum of OIPN mice after HGWD treatment. **C** The expression level of HMGB1, IL-6, TNF-α, and ZO-1 in the colon of OIPN mice treated with HGWD (magnification: 200×). Data are expressed as mean ± SEM. #P < 0.05, ##P < 0.01 (compared with Vehicle group); *P < 0.05, **P < 0.01 (compared with OIPN group)

The pharmacodynamic study found that HGWD effectively alleviates peripheral nerve pain sensitivity in chronic OIPN mice. Using IHC, we dynamically measured the amount of the relevant inflammatory factors, HMGB1, IL-6, and TNF-α, in the DRG of mice at different time periods (days 5, 10, 15, and 20). The data showed that oxaliplatin significantly elevated the expression levels of HMGB1, IL-6, and TNF-α in the DRG of mice from day 5 onwards. HGWD significantly reduced the expression levels of HMGB1, IL-6, and TNF-α inflammatory factors in the DRG of the model group mice at the late stage in a dose-dependent manner with increasing treatment time (Fig. 2A; Additional file 1: Figs. S2–S4). For chronic OIPN, the cause of increased inflammation in DRG is unclear. Therefore, we also dynamically monitored the expression levels of HMGB1, IL-6, and TNF-α in serum. A higher concentration of HMGB1, IL-6, and TNF-α was detected in the serum of the model group compared with that in the Vehicle group at different time periods (Fig. 2B). HGWD treatment significantly reduced the levels of HMGB1, IL-6, and TNF-α in the serum of the model group mice at the late stage. We also found that serum LPS levels gradually increased with increasing oxaliplatin doses, and HGWD treatment significantly reduced serum LPS levels in the later stages of the model in a dose-dependent way. The intestine is an important immune organ, and the tight junctions between intestinal epithelial cells are important to maintain the functional integrity of the intestinal barrier. Increased intestinal permeability drives LPS and related inflammatory factors into circulation and affects host health [22, 40–42]. IHC showed that HGWD treatment significantly reduced HMGB1, IL-6 and TNF-α levels in the colon of the model group mice at the late stage. In addition, oxaliplatin significantly reduced the expression level of tight junction proteins ZO-1 in the colon with the accumulation of dose, while HGWD effectively improved the expression of ZO-1 and restored intestinal barrier function in the later stages of the model with an increase in treatment time (Fig. 2C; Additional file 1: Figs. S5–S8). In summary, HGWD can alleviate chronic OIPN by reducing DRG, serum, and colon-associated inflammation and serum LPS levels and improving intestinal barrier function.

### HGWD dynamically regulates intestinal flora composition in chronic OIPN mice

**Fig. 3:**
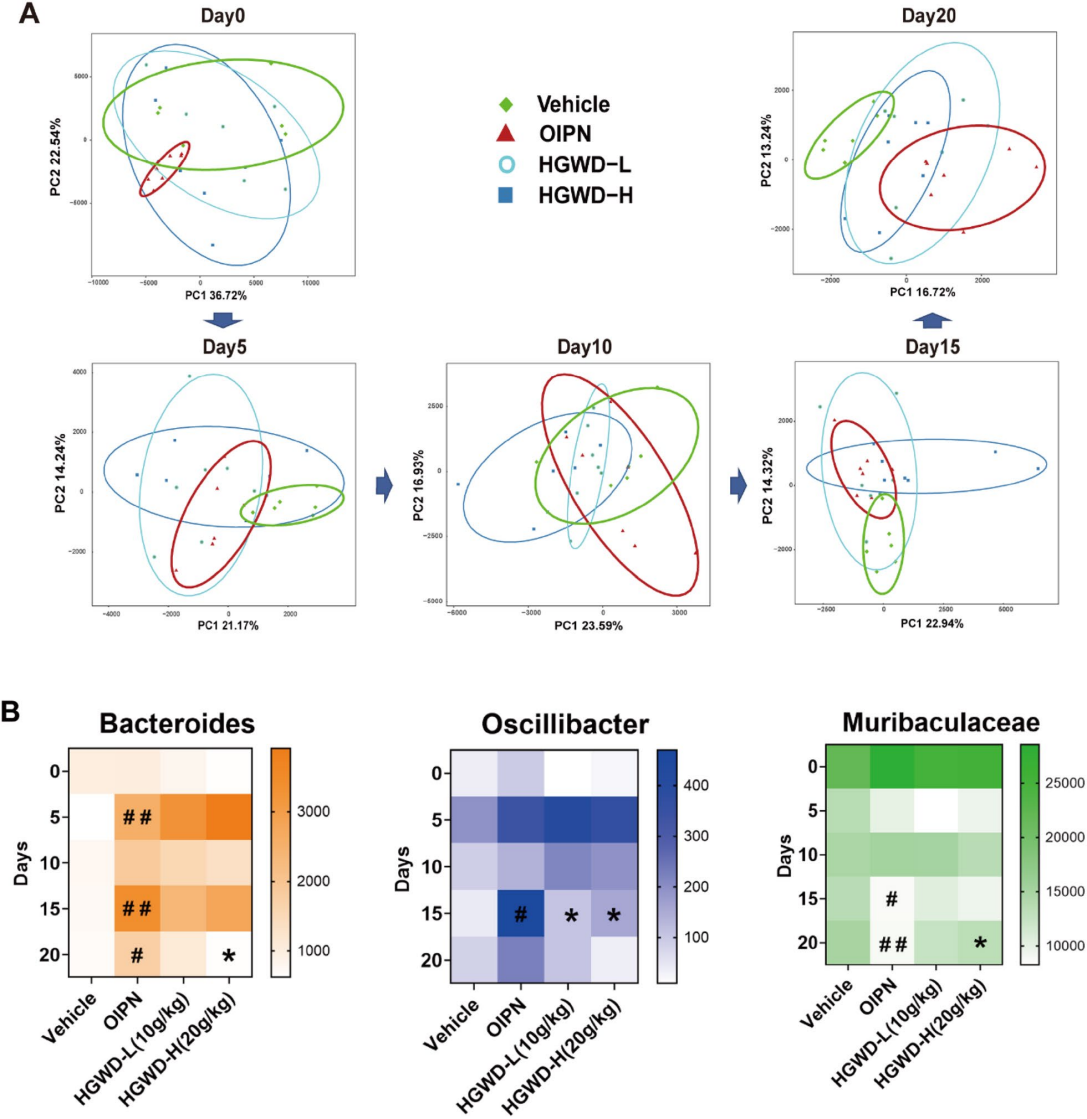
Effect of HGWD treatment on the composition of the gut microbiota in OIPN. **A** Microflora (Day 0, 5, 10, 15, and 20) PCA analysis. **B** Heat map of dynamic regulation of abundance of specific intestinal microflora, genus level. HGWD-L: 10 g/kg, HGWD-H: 20 g/kg. #P < 0.05, ##P < 0.01 (same time period, compared with Vehicle group); *P < 0.05, **P < 0.01 (same time period, compared with OIPN group)

HGWD can effectively alleviate chronic OIPN, significantly reduce the level of DRG, serum, and colon-associated inflammation, and improve intestinal barrier function in a late-stage OIPN mice model. We speculate that changes in inflammation may be related to the intestinal microbes. To investigate the role of flora in the relief of chronic pain in OIPN by HGWD, we collected fresh stool samples from mice on days 0, 5, 10, 15, and 20 and performed 16S rRNA sequencing. According to PCA (Fig. 3A), during the administration of oxaliplatin, the composition of the intestinal flora in the OIPN group gradually deviated from that in the Vehicle group and was most pronounced after two cycles of administration (Day 20). Furthermore, after the administration of high-dose HGWD and low-dose HGWD, the composition of intestinal flora showed a dynamic trend and gradually approached the Vehicle group. The data suggest that HGWD can dynamically regulate the overall composition of intestinal flora in the treatment of OIPN. According to the PCA results, we further assessed the effect of HGWD treatment on the composition of the gut flora in OIPN. First, according to the stacked histogram, 15 phyla and 142 genera were detected in all samples on the last day of the experiment (Additional file 1: Fig. S1). The phylum level mainly includes *Bacteroidota*, *Firmicutes*, *Campylobacterota*, *Desulfobacterota*, *Verrucomicrobiota*, and *Actinobaciota*; the main representative bacteria at the genus level are *Muribaculaceae*, *Lachnospiracea NK4A136*, *Ligilactobacillus*, *Lactobacillus*, and *Bacteroides*. By further dynamic detection, the composition of the intestinal microflora in the four different treatment periods on days 5, 10, 15, and 20 was determined. The data showed that oxaliplatin upregulated the harmful bacteria and downregulated the probiotics during the process of inducing peripheral neurotoxicity, whereas HGWD changed this effect after treatment (Additional file 1: Table S1). Through screening, three representative bacteria were found in the treatment of OIPN with HGWD, which showed a dynamic regulatory trend with oxaliplatin administration. As shown in Fig. 3B, after intraperitoneal injection of oxaliplatin on day 5 (just after one cycle of oxaliplatin injection, total dose 15 mg) and day 15 (just after two cycles of oxaliplatin injection, total dose 30 mg), the number of *Bacteroides* and *Oscillibacter* in the OIPN group increased significantly with the accumulation of oxaliplatin, compared to that in the Vehicle group, and bacterial abundance decreased on day 10 and day 20 (5 days after cessation of oxaliplatin administration). The abundance of *Muribaculaceae* decreased significantly with the accumulation of oxaliplatin, whereas the abundance of *Muribaculaceae* recovered on days 10 and 20 (5 days after the cessation of oxaliplatin administration); however, its self-recovery level gradually weakened with the accumulation of oxaliplatin. When HGWD treatment was administered, the abundances of *Bacteroides* and *Oscillibacter* decreased significantly, and the abundance of *Muribaculaceae* increased significantly with an increase in treatment time. The experimental data showed that HGWD alleviates the development of chronic OIPN by dynamically regulating the composition of the intestinal flora and restoring the balance of the intestinal flora.

### FMT after HGWD treatment alleviates mechanical pain and cold sensitivity in chronic OIPN mice

**Fig. 4:**
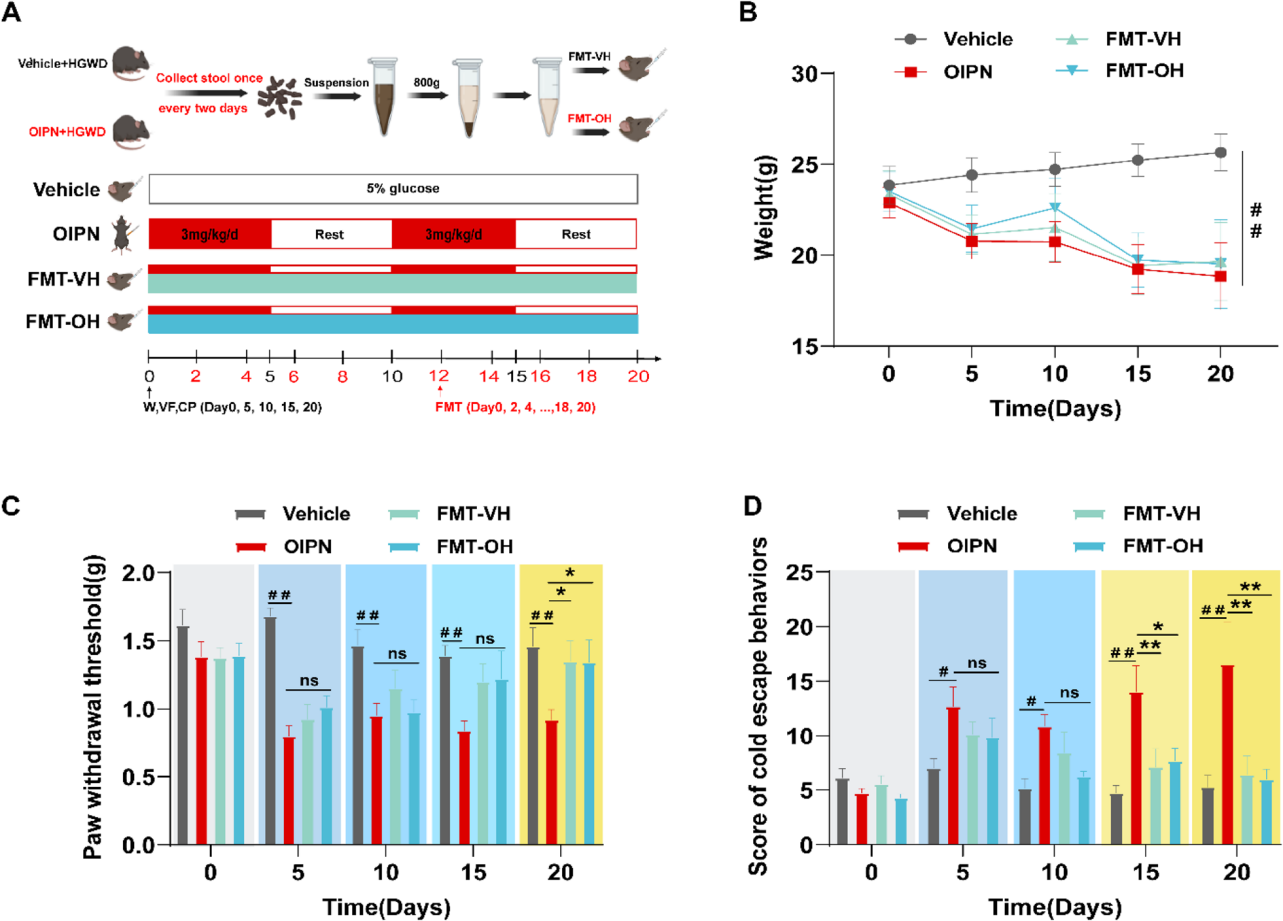
Therapeutic effect of FMT in OIPN mice. **A** The animal model of OIPN is treated with FMT after HGWD treatment. **B** Body weight test of mice. **C** Paw withdrawal threshold induced by mechanical stimulation (Von Frey filament). **D** Escape behavior score by cold stimulation (4 ℃). Data are expressed as mean ± SEM. #P < 0.05, ##P < 0.01 (compared with Vehicle group); *P < 0.05, **P < 0.01 (compared with OIPN group)

To investigate whether the intestinal flora altered by HGWD treatment had a protective effect on OIPN mice, we collected the feces of normal mice and OIPN mice after HGWD treatment to perform FMT treatment in OIPN mice (Fig. 4A). The data showed that oxaliplatin administration significantly reduced the weight of mice; however, the FMT after HGWD treatment did not restore the body weight of OIPN mice (Fig. 4B). Moreover, both FMT protocols significantly reduced high cold pain escape scores and increased mechanical pain thresholds in chronic OIPN mice compared to those in the OIPN group after two cycles of treatment (Fig. 4C, D). The experimental data showed that HGWD effectively alleviated oxaliplatin-induced chronic peripheral nerve mechanical pain and cold pain sensitivity by restoring intestinal flora homeostasis.

### FMT after HGWD treatment reduced the intestinal, serum, and DRG inflammation infiltration in OIPN mice

**Fig. 5:**
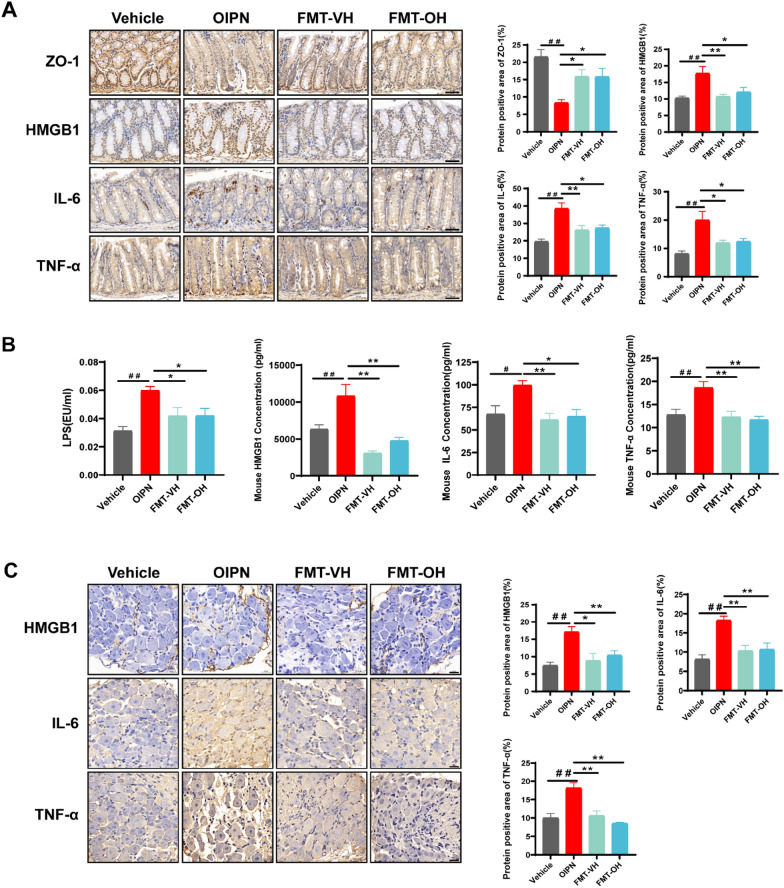
Effect of FMT on intestinal barrier, serum LPS, and inflammation in colon, serum, and DRG in OIPN mice. **A** Expression level of ZO-1, HMGB1, IL-6, and TNF-α in the colon of OIPN mice after FMT (magnification: 200×). **B** Relative levels of LPS, HMGB1, IL-6, and TNF-α in the serum of OIPN mice after FMT. **C** Expression level of HMGB1, IL-6, and TNF-α in the DRG of OIPN mice after FMT (magnification: 500×). Data are expressed as mean ± SEM. #P < 0.05, ##P < 0.01 (compared with Vehicle group); *P < 0.05, **P < 0.01 (compared with OIPN group)

Figure 5A show that the FMT of HGWD treatment normal mice and OIPN mice increased ZO-1 expression and significantly decreased the expression levels of HMGB1, IL-6, and TNF-α in the colon compared with that in the Vehicle group. Further, we detected the level of LPS in serum and IL-6, TNF-α, and HMGB1 in serum and DRG of OIPN mice. The data showed that the FMT of HGWD treatment could significantly reduce the level of IL-6, TNF-α, and HMGB1 expression in serum and DRG and significantly reduce the level of LPS in serum (Fig. 5B, C). The experimental data showed that FMT after HGWD treatment restores the intestinal barrier’s functional integrity and decreases the content of serum LPS and the expression of related inflammatory markers in the colon, serum, and DRG to prevent OIPN, which plays the same role as that of HGWD treatment.

### ABX treatment partially eliminates the protective effect of HGWD on OIPN mice

**Fig. 6:**
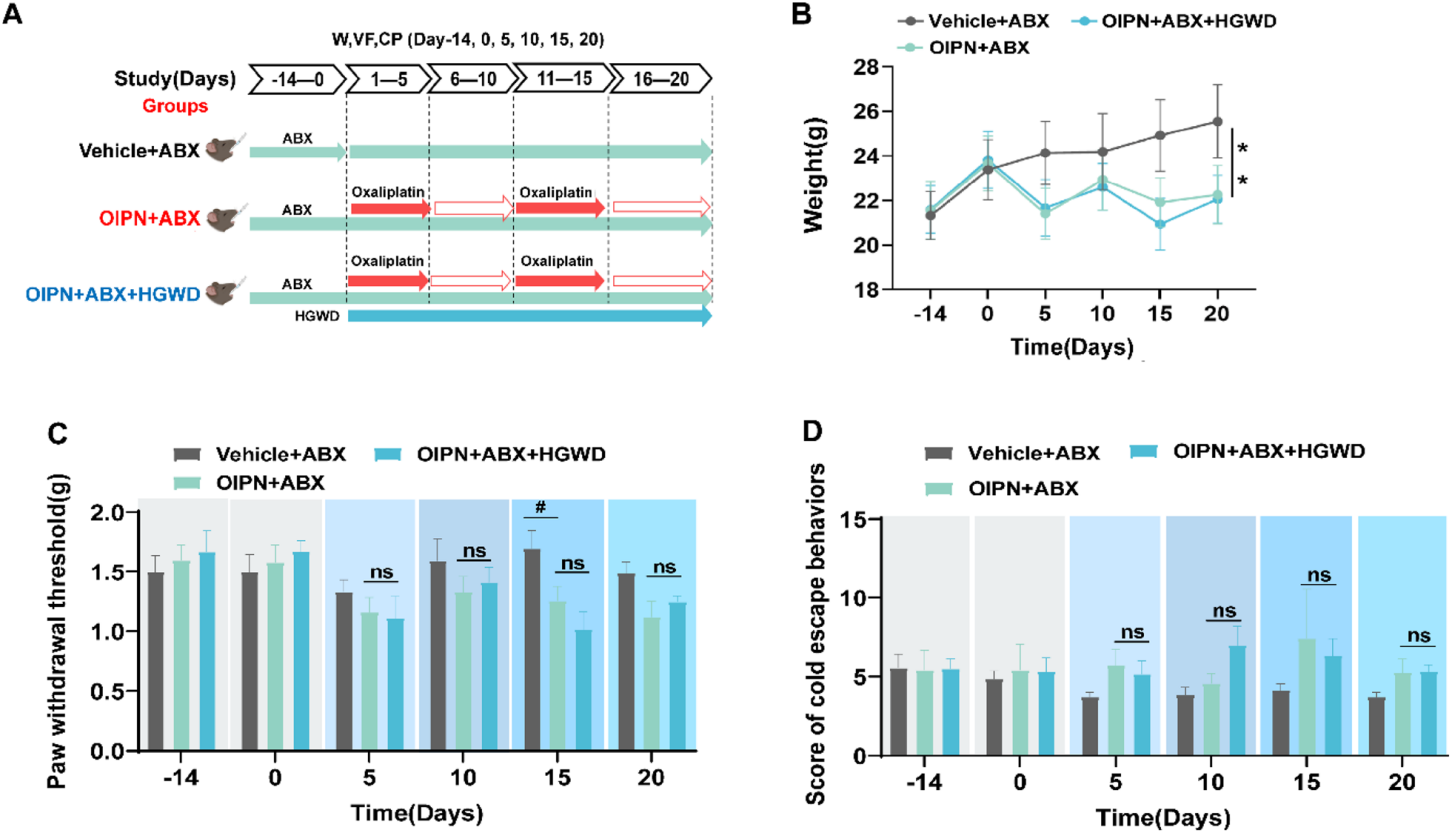
The role of antibiotic pretreatment validation flora in HGWD treatment of OIPN. **A** Under the ABX condition, HGWD is used to treat oxaliplatin-induced peripheral neurotoxicity (OIPN) animal model. **B** Body weight test of mice. **C** Foot retraction threshold by mechanical stimulation (Von Frey filament). **D** Escape behavior score by cold stimulation (4 °C). Data are expressed as mean ± SEM. #P < 0.05, ##P < 0.01 (compared with Vehicle group); *P < 0.05, **P < 0.01 (compared with OIPN group)

To further verify the beneficial role of flora in the treatment of OIPN by HGWD, we established a pseudo germ-free mouse model using ABX and treated it with HGWD (Fig. 6A). After ABX cleared most of the intestinal microflora, oxaliplatin administration significantly reduced the weight of the mice, whereas HGWD treatment did not restore the weight of the mice (Fig. 6B). In addition, under ABX intervention, HGWD treatment did not effectively alleviate pain sensitivity in OIPN mice (Fig. 6C, D). Experimental data show that intestinal flora is important for HGWD treatment of OIPN.

## Discussion

A variety of drugs have been reported to prevent and treat chemotherapy-induced peripheral neurotoxicity, but there are no recommended drugs with good efficacy against chronic chemotherapy-induced neurotoxicity because of their obvious toxic side effects and lack of significant clinical efficacy during treatment [9]. Chinese medicine is a promising multi-target and multi-pathway synergistic intervention for the prevention of OIPN. HGWD can effectively prevent the occurrence of OIPN and has a high safety profile. Our results show that HGWD treatment can effectively alleviate oxaliplatin-induced chronic peripheral nerve pain response, significantly reduce serum LPS, and DRG, serum, and colon-associated inflammation levels, and improve intestinal barrier function in the late stages of the model. 16S rRNA sequencing results showed that HGWD treatment dynamically restored oxaliplatin-induced gut microbial dysbiosis. ABX and FMT experiments showed that the intestinal flora is important for HGWD treatment of chronic OIPN and that HGWD can treat chronic OIPN by dynamically restoring intestinal flora homeostasis, with HGWD playing the same role.

Trillions of microorganisms colonize our intestinal tract. Under normal conditions, the gut microbiota is in dynamic balance. However, when stimulated by external factors, the balance of the intestinal microbiota is disturbed, leading to various diseases [34, 43–46]. Therefore, the interaction between homeostatic regulation of the organism and intestinal flora is a key requirement for host health [47]. According to 16S RNA sequencing dynamic analysis, HGWD treatment improved intestinal dysbiosis induced by oxaliplatin in mice, which was manifested by the dynamic restoration of the overall composition of the intestinal flora and the number of associated flora. Previous studies have reported that among the relevant herbal components that make up HGWD, astragali polysaccharides and astragali saponins in astragalus, cinnamaldehyde in cinnamon sticks, paeoniflorin in peony, jujube polysaccharides in jujube, ginger polysaccharides in ginger, and exosome nanoparticles can be used in the treatment of the disease by regulating the composition of the intestinal flora and improving the dysfunction of intestinal bacterial homeostasis [48–53]. Therefore, we believe that the therapeutic effect of HGWD on OIPN is mainly due to remodeling of intestinal microbial homeostasis. The FMT results demonstrated that restoring intestinal microbiota homeostasis in OIPN mice significantly relieved pain sensitivity. At the genus level, we further screened using dynamic analysis and found that the three representative bacteria (*Bacteroides*, *Oscillibacter*, and *Muribaculaceae*) demonstrated a dynamic regulatory trend with oxaliplatin administration in the HGWD treatment of OIPN. The data showed that as the dose of oxaliplatin administered increased, the number of *Bacteroides* and *Oscillibacter* significantly increased and the number of *Muribaculaceae* bacteria significantly decreased in the OIPN group. After HGWD treatment, there was a significant decrease in the number of *Bacteroides* and *Oscillibacter* and a significant increase in the number of *Muribaculaceae* with the increase in treatment time. *Bacteroides* is a gram-negative anaerobe found in the intestine that increases the production of inflammatory factors in the colon [54]. Research shows that the removal of intestinal microflora can significantly reduce mechanical pain caused by chemotherapy; when the microflora is restored, the protective effects disappears. Further analysis of the abundance of *Bacteroides* in the feces revealed that it was restored or even elevated in the feces of transplanted mice and was positively correlated with the degree of pain behavior [55]. Our results also demonstrated that HGWD treatment of OIPN mice significantly reduced the abundance of *Bacteroides*, which were upregulated by oxaliplatin. This indicates that it is important in the development of OIPN, and preventing its abundance may be an effective method for treating OIPN. In subsequent experiments, the effect of *Bacteroides* on the development of OIPN and the mechanism of action should be explored in depth. *Oscillibacter* is increased in intestinal inflammation and related diseases [45, 56]. There is evidence that *Muribaculaceae* can improve intestinal mucositis [46, 57]. Research shows that the potential mechanism of PRS alleviation of the inflammatory response may be related to an increase in *Murbaculaceae* [34]. Therefore, we need to further explore the role of related bacteria in the development of OIPN in future research.

Inflammation is important for nerve pain caused by peripheral nerve injury [58, 59]. As a result of peripheral nerve injury, the local inflammatory response increases neuropathic pain [60]. We found that during the treatment of chronic OIPN, both high and low doses of HGWD significantly reduced the expression of IL-6, TNF-α, and HMGB1 in the DRG, serum, and colon of the model group mice at the late stage, with the prolongation of the treatment period. These data suggest that the prevention of chronic OIPN by HGWD is closely related with the inhibition of associated inflammation in the DRG, serum, and colon of the model group mice at the late stage. However, as we selected the effective clinical doses of HGWD for the treatment of OIPN (10 g/kg and 20 g/kg), which were proven in the group in the previous period, the relevant data showed that there was no statistically significant difference between high and low doses of HGWD treatment, although both of them exerted significant therapeutic effects on chronic OIPN and showed a certain dose-dependent trend. We will further reduce or increase the dose of HGWD for dynamic study in the future. There is growing evidence that intestinal flora and their metabolites can alter the structure and contribute to disease improvement by reshaping the intestinal immune microenvironment through probiotic use or microbiota transplantation [61]. Our study demonstrated that FMT after HGWD treatment significantly alleviated the peripheral nerve pain response in chronic OIPN and reduced the expression levels of IL-6, TNF-α, and HMGB1 in the DRG, serum, and colon of the model group mice at the late stage. Research has shown that HMGB1 can readily initiate an inflammatory response in concert with other inflammatory factors, and drugs that inhibit HMGB1 production and anti-HMGB1 antibodies are associated with a reduction in neuroinflammation [22, 62]. It is also important for the development of chemotherapy-induced pain. HGWD treatment significantly suppressed HMGB1 expression levels in the later stages of the model, and FMT proved to be associated with the restoration of intestinal flora homeostasis. We also found that by prolonging the HGWD treatment time, the damage to the intestinal barrier function caused by oxaliplatin administration could be gradually improved. The serum LPS levels were also reduced. Recent studies have found that mechanical hyperalgesia in mice with OIPN is reduced after antibiotic pretreatment, and this protective effect may be due to the elimination of LPS. Indeed, exogenous LPS can abolish the protection provided by eradication of the gut microbiota [11]. FMT has similarly demonstrated that restored gut flora improves gut barrier function and reduces serum LPS levels. When intestinal permeability is altered, LPS enters the circulation and promotes the production of a number of pro-inflammatory factors. Therefore, we conjectured that the alteration of LPS in serum might be associated with the alteration of intestinal barrier integrity. We need to verify this in future experiments. HGWD can alleviate OIPN by regulating the balance of intestinal flora and inhibiting the expression of associated inflammation, and possibly also by affecting neurotransmitters and associated glial cells. We intend to explore this in future experiments.

Chinese medicines are characterized by multicomponents and multipathways; therefore, the study of chemical composition of chinese medicines is important. Our group has also pre-screened the major HGWD compounds [29]. We are currently investigating how HGWD-related compounds modulate relevant ion channels to alleviate OIPN. Moreover, our previous study found the beneficial role of formononetin (FN) as a major component of Astragalus extract in preventing OIPN-induced mitochondrial dysfunction and apoptosis [39]. In subsequent experiments, we will continue to study the interactions between the major compounds in the herbs and the gut flora and their metabolism. This future work will serve as an extension of this study in the direction of modern research in Chinese medicine. The linkage and temporal sequence of changes in the dynamics of associated inflammation from the colon, serum, and DRG and the specific bacteria that play a role in HGWD for chronic OIPN and their mechanisms identified in this study need to be further explored.

## Conclusion

**Fig. 7:**
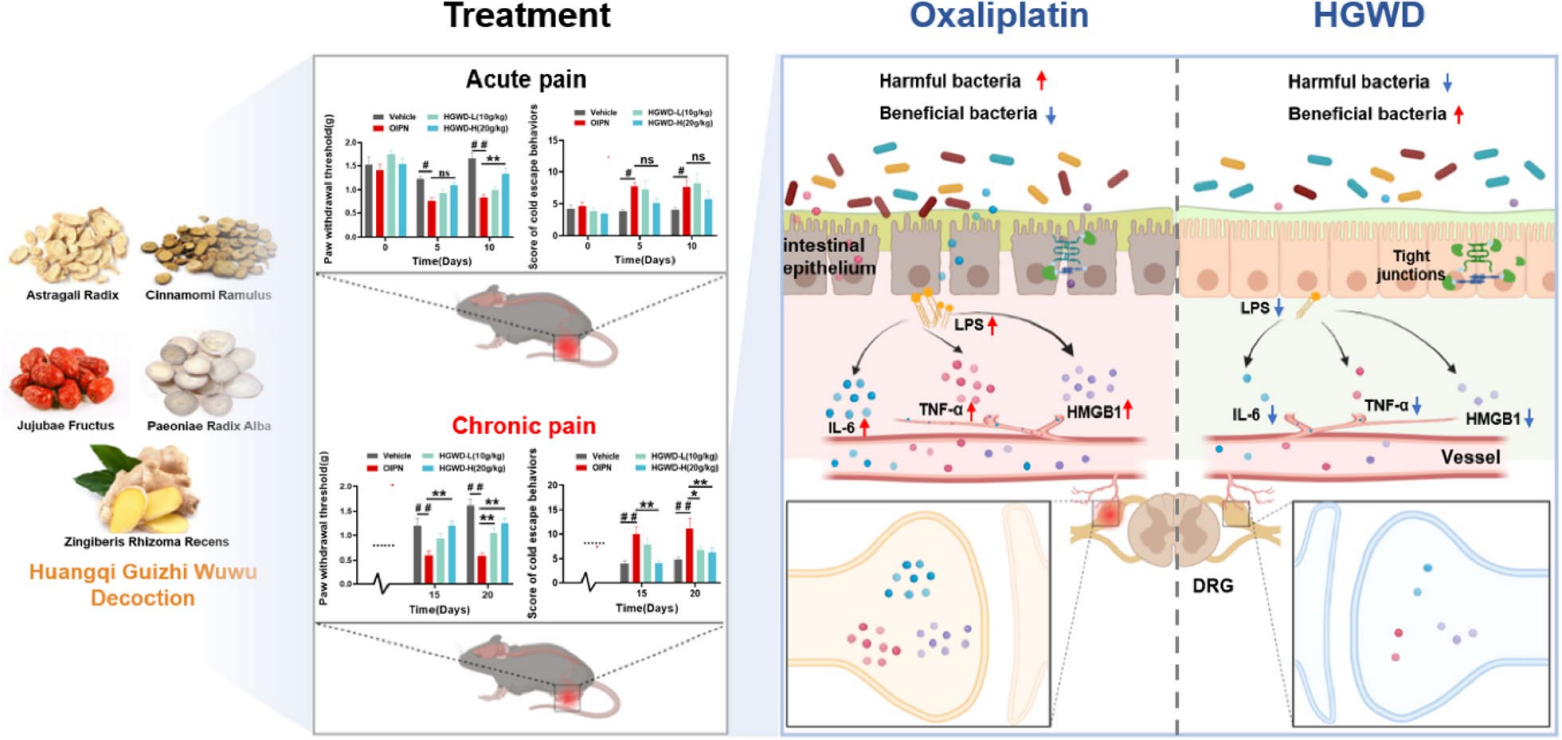
Mechanistic map of HGWD for the prevention of chronic OIPN.

In summary, our findings suggest that oral HGWD can prevent chronic OIPN by dynamically regulating intestinal flora homeostasis, improving the integrity of the intestinal barrier function, and reducing LPS accumulation in the serum and the levels of associated inflammatory proteins in the colon, serum, and DRG (Fig. 7). These findings provide a rationale for the role of homeostatic regulation of intestinal flora in the treatment of OIPN by HGWD and offer hope for the application of microbial therapy for OIPN.

### Supplementary Information

**Additional file 1: Table S1**. Effect of HGWD on intestinal microflora of OIPN mice. **Fig. S1**. Effect of HGWD treatment on the gut flora composition of OIPN. **Fig. S2**. Effect of HGWD treatment on the expression level of HMGB1 in the DRG at different time periods. **Fig. S3**. Effect of HGWD treatment on the expression level of IL-6 in the DRG at different time periods. **Fig. S4**. Effect of HGWD treatment on the expression level of TNF-α in the DRG at different time periods. **Fig. S5**. Effect of HGWD treatment on the expression level of ZO-1 in the colon at different time periods. **Fig. S6**. Effect of HGWD treatment on the expression level of HMGB1 in the colon at different time periods. **Fig. S7**. Effect of HGWD treatment on the expression level of IL-6 in the colon at different time periods. **Fig. S8**. Effect of HGWD treatment on the expression level of TNF-α in the colon at different time periods.

**Additional file 2. Figure S9**. Representative base peak intensity (BPI) chromatograms of HGWD analyzed by LC-MS in positive (A) and negative (B) mode. **Table S2**. Components of HGWD identified by LC-MS. **Table S3**. Calibration curves, limit of detection (LOD), limit of quantification (LOQ), accuracy, recovery, and contents of six analytes in HGWD. **Table Sa4**. Active compounds and their corresponding parameters in the HGWD formula.

## Data Availability

In this published article, all data generated or analyzed during this study are included (and its Additional files). Source data are also provided.

## Abbreviations

ABX: Antibiotic treatment
ANOVA: Analysis of variance
CIPN: Chemotherapy-induced peripheral neuropathy
CP: Cold pain score
DRG: Dorsal root ganglion
FC: Mouse feces collection
FMT: Fecal microbiota transplantation
FMT-OH: Fecal treatment of OIPN mice treated with HGWD transplantation
FMT-VH: Fecal treatment of Vehicle mice treated with HGWD transplantation
HGWD: Huangqi Guizhi Wuwu Decoction
HGWD-L: Huangqi Guizhi Wuwu Decoction low-dose
HGWD-H: Huangqi Guizhi Wuwu Decoction high-dose
HMGB1: High mobility group box 1
LPS: Lipopolysaccharide
OIPN: Oxaliplatin-induced peripheral neurotoxicity
PCA: Principal component analysis
SEM: Standard error of the mean
TCM: Traditional Chinese Medicine
VF: Mechanical pain measurement (Von Frey filament)
W: Body weight
