# Supplementary material for: Huangqi Guizhi Wuwu decoction alleviates oxaliplatin-induced peripheral neuropathy via the gut-peripheral nerve axis
Source: Chin Med. 2023 Sep 7;18:114. doi: 10.1186/s13020-023-00826-5 (PMC10485938; doi:10.1186/s13020-023-00826-5)
Supplement: Supplementary file 1 — Additional file 1: Table S1. Effect of HGWD on intestinal microflora of OIPN mice. Fig. S1. Effect of HGWD treatment on the gut flora composition of OIPN. Fig. S2. Effect of HGWD treatment on the expression level of HMGB1 in the DRG at different time periods. Fig. S3. Effect of HGWD treatment on the expression level of IL-6 in the DRG at different time periods. Fig. S4. Effect of HGWD treatment on the expression level of TNF-α in the DRG at different time periods. Fig. S5. Effect of HGWD treatment on the expression level of ZO-1 in the colon at different time periods. Fig. S6. Effect of HGWD treatment on the expression level of HMGB1 in the colon at different time periods. Fig. S7. Effect of HGWD treatment on the expression level of IL-6 in the colon at different time periods. Fig. S8. Effect of HGWD treatment on the expression level of TNF-α in the colon at different time periods. [file 13020_2023_826_MOESM1_ESM.docx]

**Additional file 1**

Table S1. Effect of HGWD on gut microbiota of OIPN mice.

| **Genus (Vehicle group vs. OIPN group)** | | | | | **HGWD group** |
| --- | --- | --- | --- | --- | --- |
| **Abundance** | **Day 5** | **Day 10** | **Day 15** | **Day 20** | **Abundance** |
| Up-  regulated | Escherichia-Shigella | Escherichia-Shigella | Escherichia-Shigella | Escherichia-Shigella | Down-  regulated |
|  | Oscillibacter | Oscillibacter | Oscillibacter | Oscillibacter |  |
|  | Bacteroides | Bacteroides | Bacteroides | Bacteroides |  |
|  | Helicobacter | Helicobacter | Helicobacter | Helicobacter |  |
|  | Acetatifactor | / | / | / |  |
|  | Ruminococcus | / | / | / |  |
|  | Mycoplasma | / | / | / |  |
|  | / | Lachnospiraceae UCG-001 | / | / |  |
|  | / | Anaerostipes | / | / |  |
|  | / | / | Desulfovibrio | / |  |
|  | / | / | Colidextribacter | / |  |
|  | / | / | Blautia | / |  |
|  | / | / | / | Alistipes |  |
|  | / | / | / | Odoribacter |  |
|  | / | / | / | Gastranaerophilales_norank |  |
|  | | | | | |
| Down-  regulated | Dubosiella | Dubosiella | Dubosiella | Dubosiella | Up-  regulated |
|  | Lactobacillus | Lactobacillus | Lactobacillus | Lactobacillus |  |
|  | Muribaculaceae  _norank | Muribaculaceae  _norank | Muribaculaceae  _norank | Muribaculaceae  _norank |  |
|  | Ligilactobacillus | / | / | / |  |
|  | Prevotellaceae UCG-001 | / | / | / |  |
|  | / | Prevotellaceae NK3B31 group | / | / |  |
|  | / | LachnospiraceaeNK4A136 group | / | / |  |
|  | / | / | Limosilactobacillus | / |  |
|  | / | / | Bifidobacterium | / |  |
|  | / | / | / | Butyrivibrio |  |
|  | / | / | / | Coriobacteriaceae UCG-002 |  |

Table S1. Statistical analysis of the gut microbiota composition at four different time points of OIPN treatment with HGWD on days 5, 10, 15, and 20 is performed at the genus level. Abbreviations: HGWD, Huangqi Guizhi Wuwu Decoction; OIPN, oxaliplatin-induced peripheral neurotoxicity.


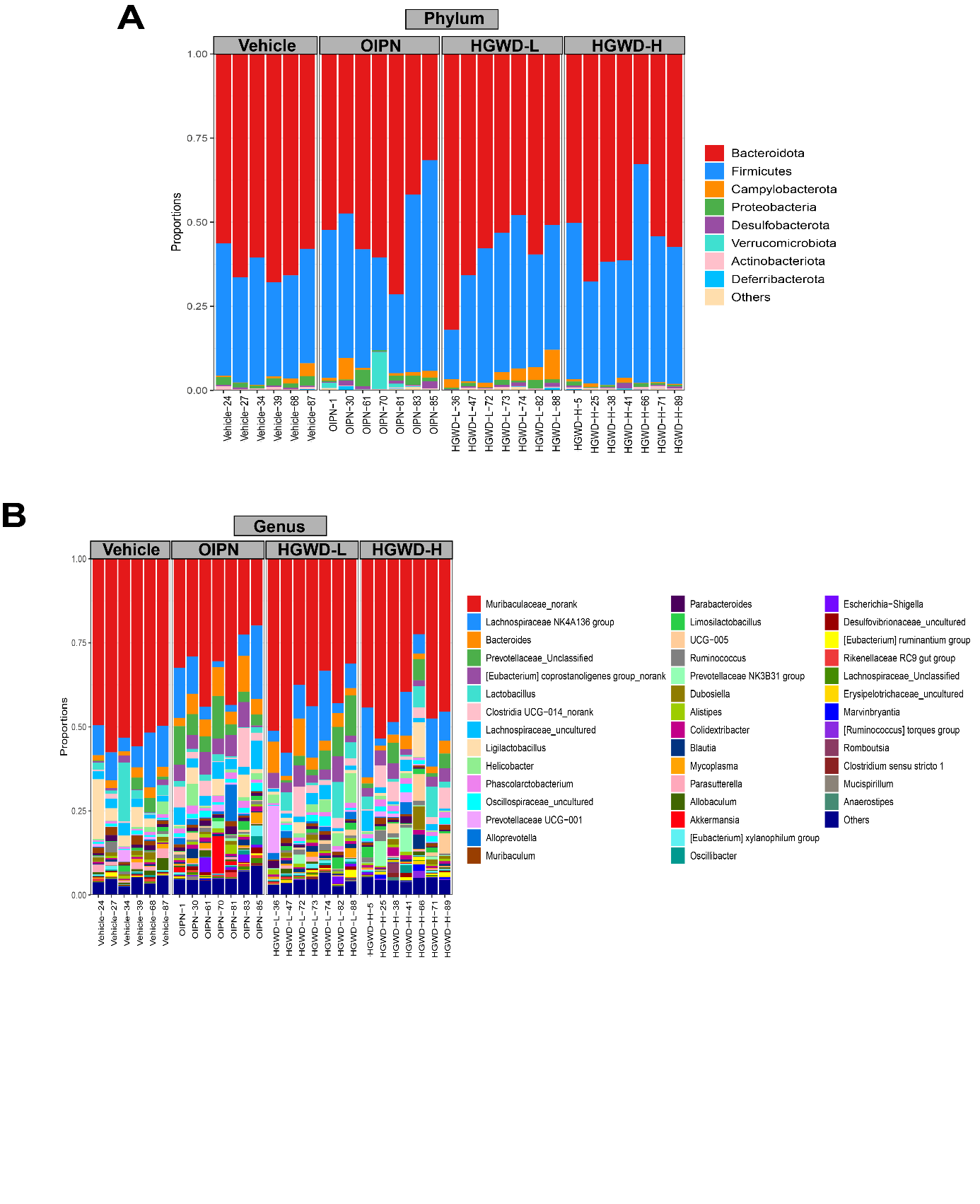


Figure S1. Effect of HGWD treatment on the gut microbiota composition of OIPN mice. (A) Histogram of the gut microbiota pile, at the phylum level. (B) Histogram of the gut microbiota pile, at the genus level. Abbreviations: HGWD-L, Huangqi Guizhi Wuwu Decoction low-dose; HGWD-H, Huangqi Guizhi Wuwu Decoction high-dose; OIPN, oxaliplatin-induced peripheral neurotoxicity.


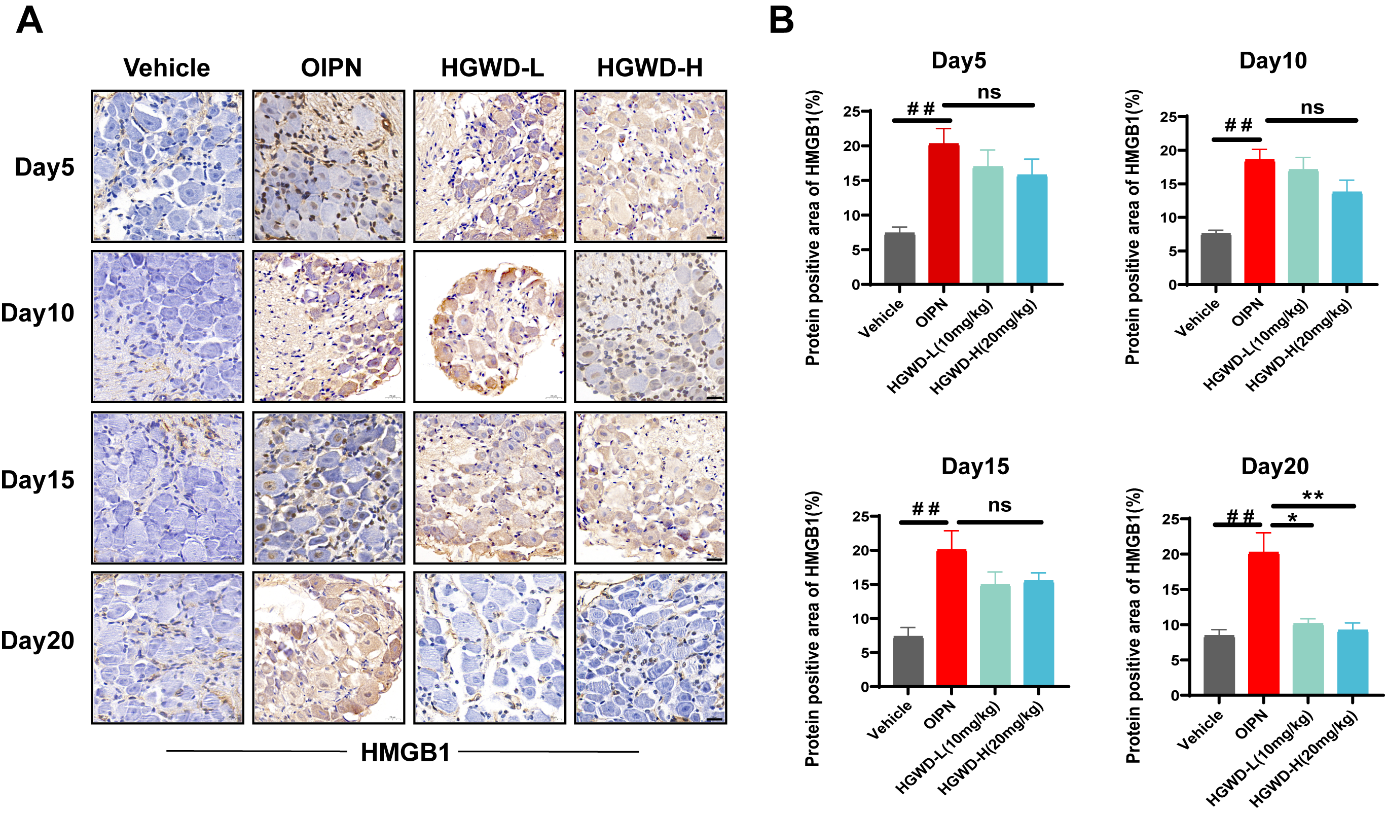


Figure S2. Effect of HGWD treatment on the expression level of HMGB1 in the DRG at different time periods (500×). (A) DRG HMGB1 immunohistochemistry; (B) The expression level of HMGB1 in the DRG. # P<0.05, ## P<0.01 (compared with Vehicle group); * P<0.05, ** P<0.01 (compared with OIPN group). Abbreviations: HGWD-L, Huangqi Guizhi Wuwu Decoction low-dose; HGWD-H, Huangqi Guizhi Wuwu Decoction high-dose; OIPN, oxaliplatin-induced peripheral neurotoxicity; ns, not significant; DRG, dorsal root ganglion.


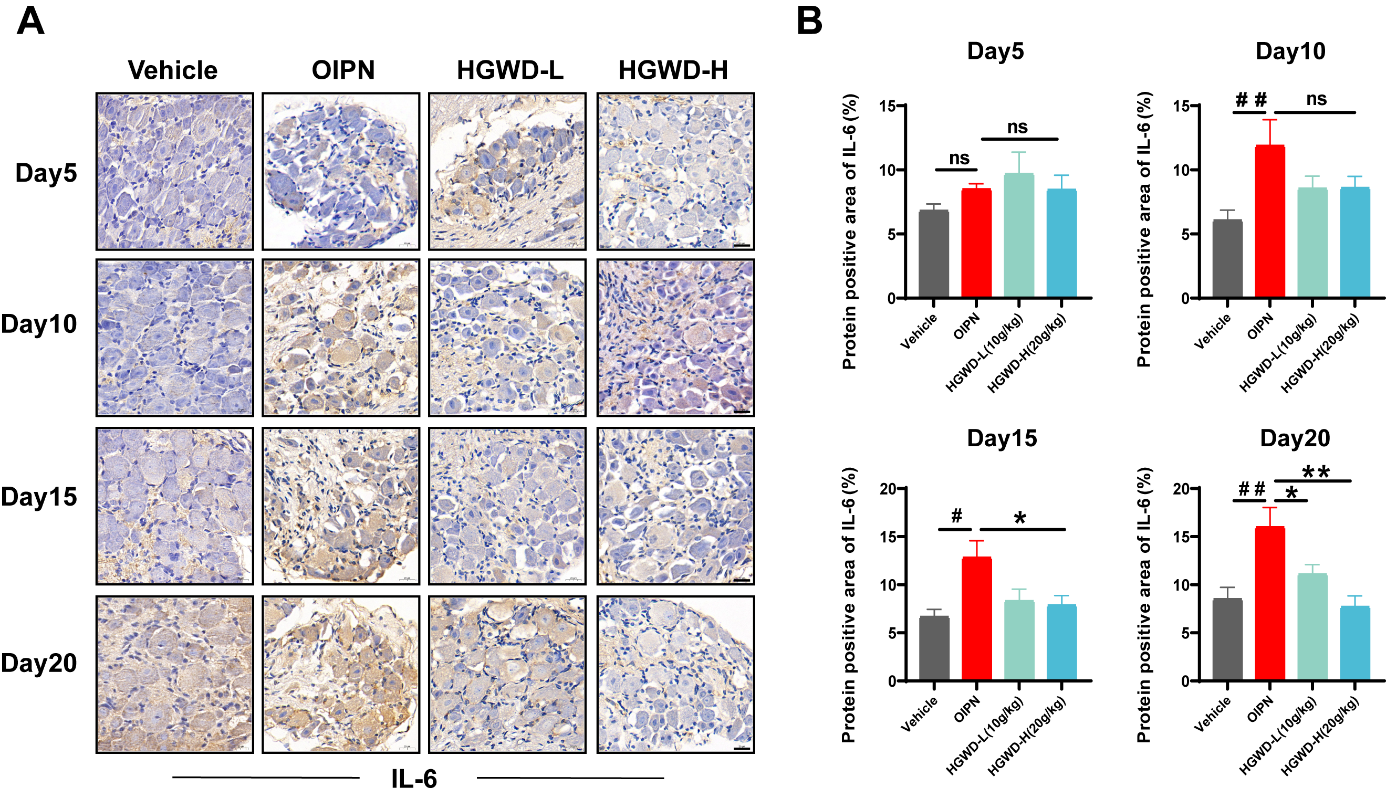


Figure S3. Effect of HGWD treatment on the expression level of IL-6 in the DRG at different time periods (500×). (A) DRG IL-6 immunohistochemistry; (B) The expression level of IL-6 in the DRG. # P<0.05, ## P<0.01 (compared with Vehicle group); * P<0.05, ** P<0.01 (compared with OIPN group). Abbreviations: HGWD-L, Huangqi Guizhi Wuwu Decoction low-dose; HGWD-H, Huangqi Guizhi Wuwu Decoction high-dose; OIPN, oxaliplatin-induced peripheral neurotoxicity; ns, not significant; DRG, dorsal root ganglion.


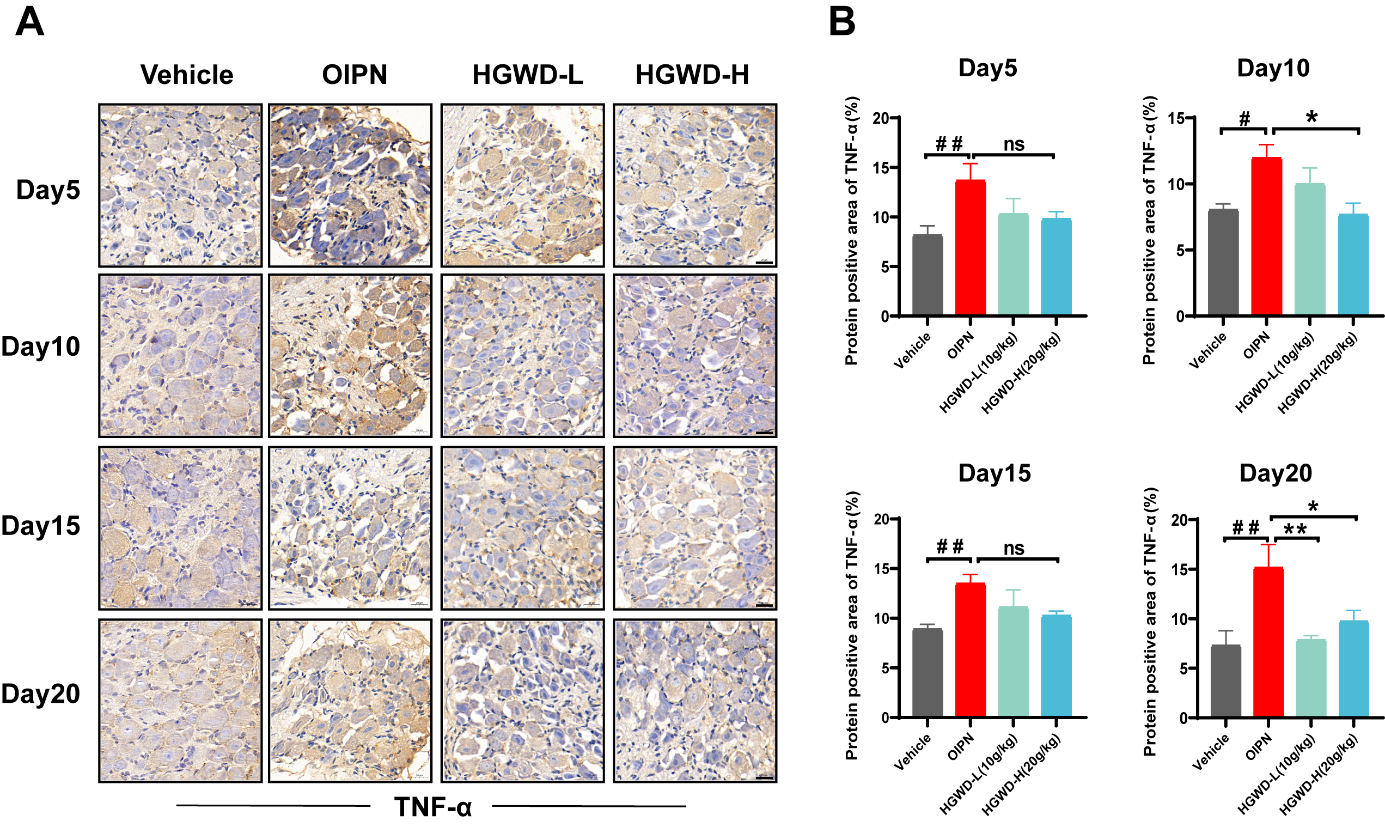


Figure S4. Effect of HGWD treatment on the expression level of TNF-α in the DRG at different time periods (500×). (A) DRG TNF-α immunohistochemistry; (B) The expression level of TNF-α in the DRG. # P<0.05, ## P<0.01 (compared with Vehicle group); * P<0.05, ** P<0.01 (compared with OIPN group). Abbreviations: HGWD-L, Huangqi Guizhi Wuwu Decoction low-dose; HGWD-H, Huangqi Guizhi Wuwu Decoction high-dose; OIPN, oxaliplatin-induced peripheral neurotoxicity; DRG, dorsal root ganglion; ns, not significant.


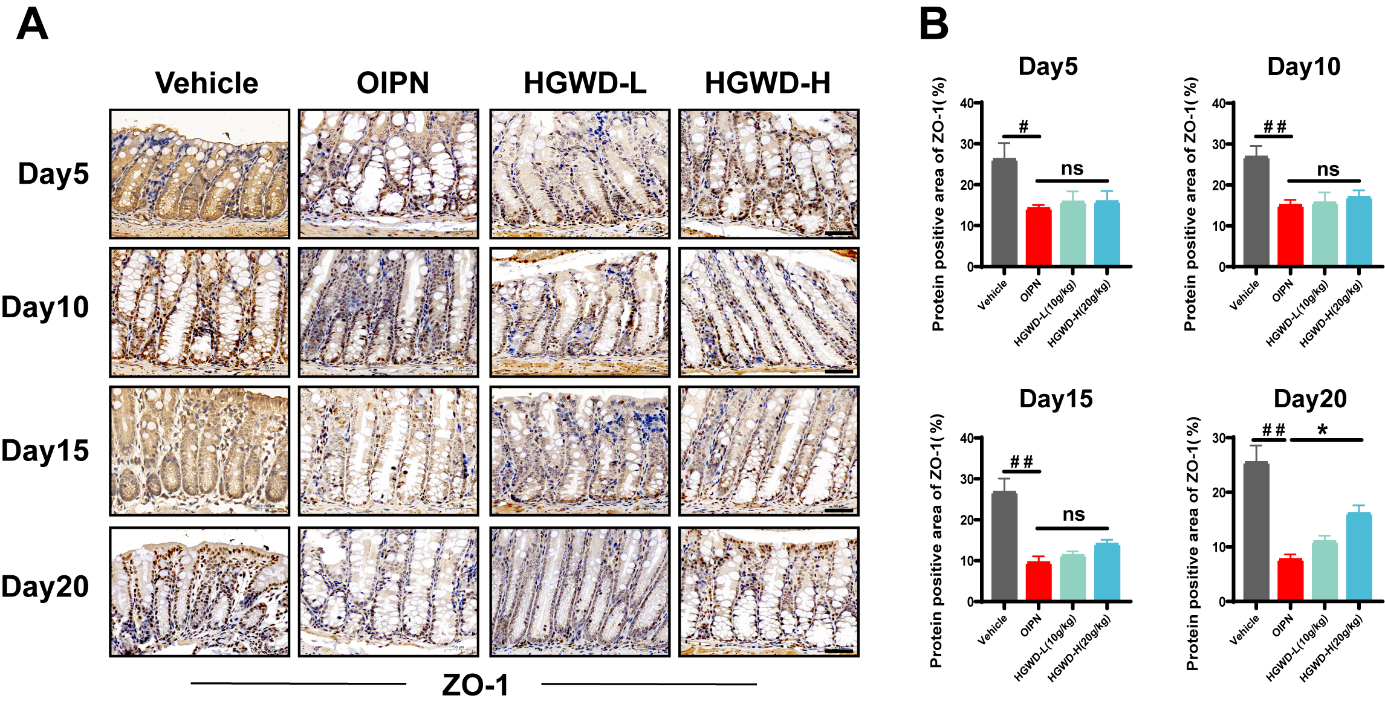


Figure S5. Effect of HGWD treatment on the expression level of ZO-1 in the colon at different time periods (200×). (A) Colon ZO-1 immunohistochemistry; (B) Expression levels of the intestinal tight junction protein ZO-1. # P<0.05, ## P<0.01 (compared with Vehicle group); * P<0.05, ** P<0.01 (compared with OIPN group). Abbreviations: HGWD-L, Huangqi Guizhi Wuwu Decoction low-dose; HGWD-H, Huangqi Guizhi Wuwu Decoction high-dose; OIPN, oxaliplatin-induced peripheral neurotoxicity; ns, not significant.


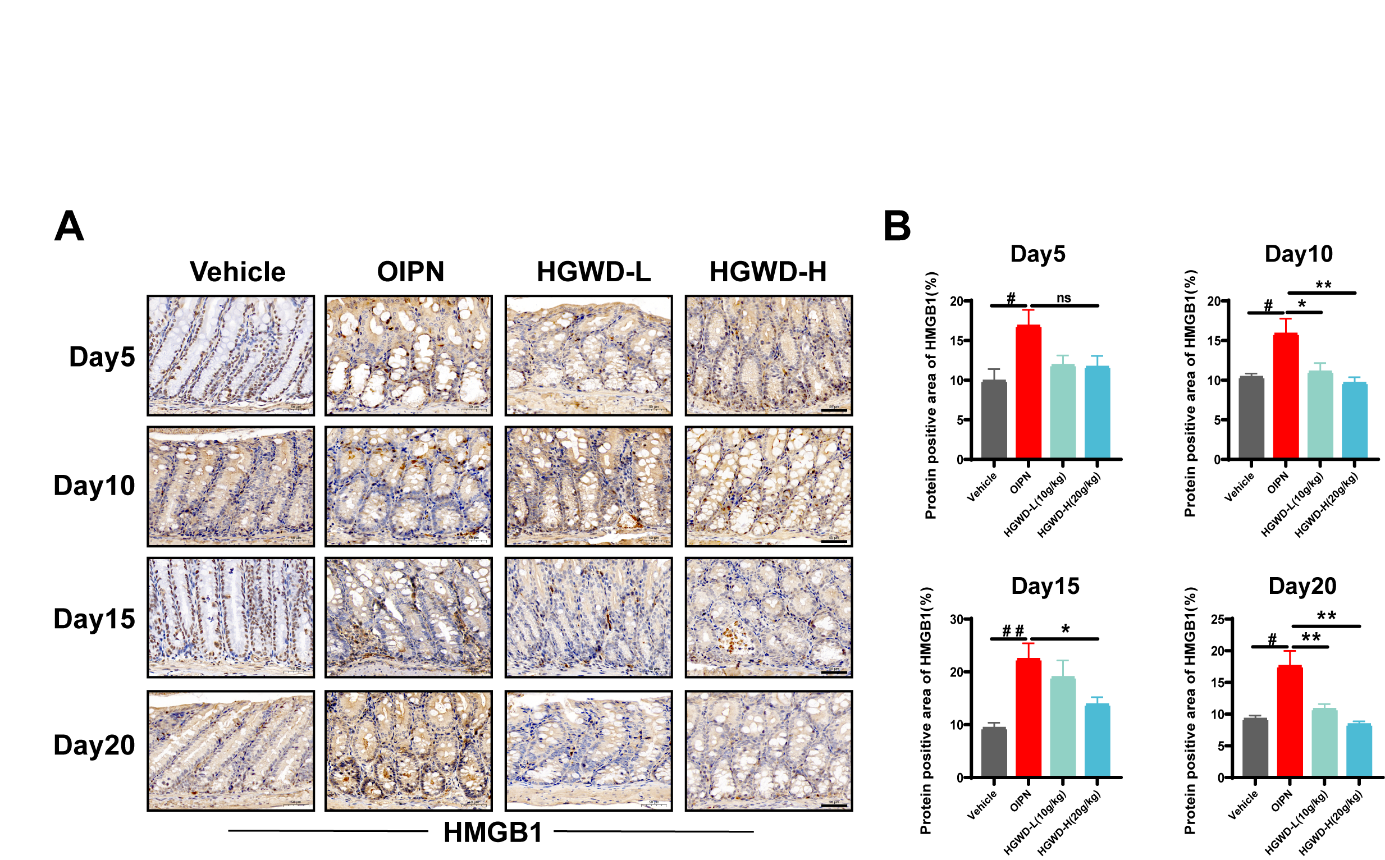


Figure S6. Effect of HGWD treatment on the expression level of HMGB1 in the colon at different time periods (200×). (A) Colon HMGB1 immunohistochemistry; (B) The expression level of HMGB1 in the colon. # P<0.05, ## P<0.01 (compared with Vehicle group); * P<0.05 ** P<0.01 (compared with OIPN group). Abbreviations: HGWD-L, Huangqi Guizhi Wuwu Decoction low-dose; HGWD-H, Huangqi Guizhi Wuwu Decoction high-dose; OIPN, oxaliplatin-induced peripheral neurotoxicity; ns, not significant.


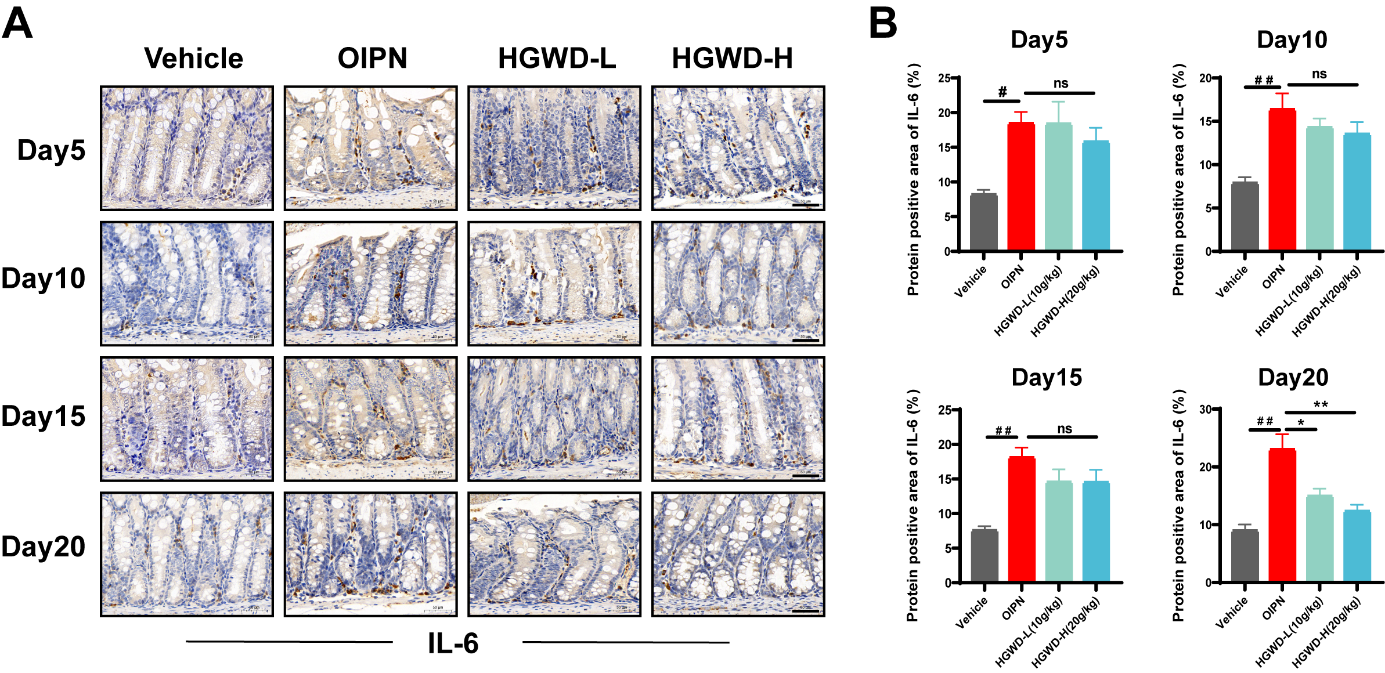


Figure S7. Effect of HGWD treatment on the expression level of IL-6 in the colon at different time periods (200×). (A) Colon IL-6 immunohistochemistry; (B) The expression level of IL-6 in the colon. # P<0.05, ## P<0.01 (compared with Vehicle group); * P<0.05, ** P<0.01 (compared with OIPN group). Abbreviations: HGWD-L, Huangqi Guizhi Wuwu Decoction low-dose; HGWD-H, Huangqi Guizhi Wuwu Decoction high-dose; OIPN, oxaliplatin-induced peripheral neurotoxicity; ns, not significant.


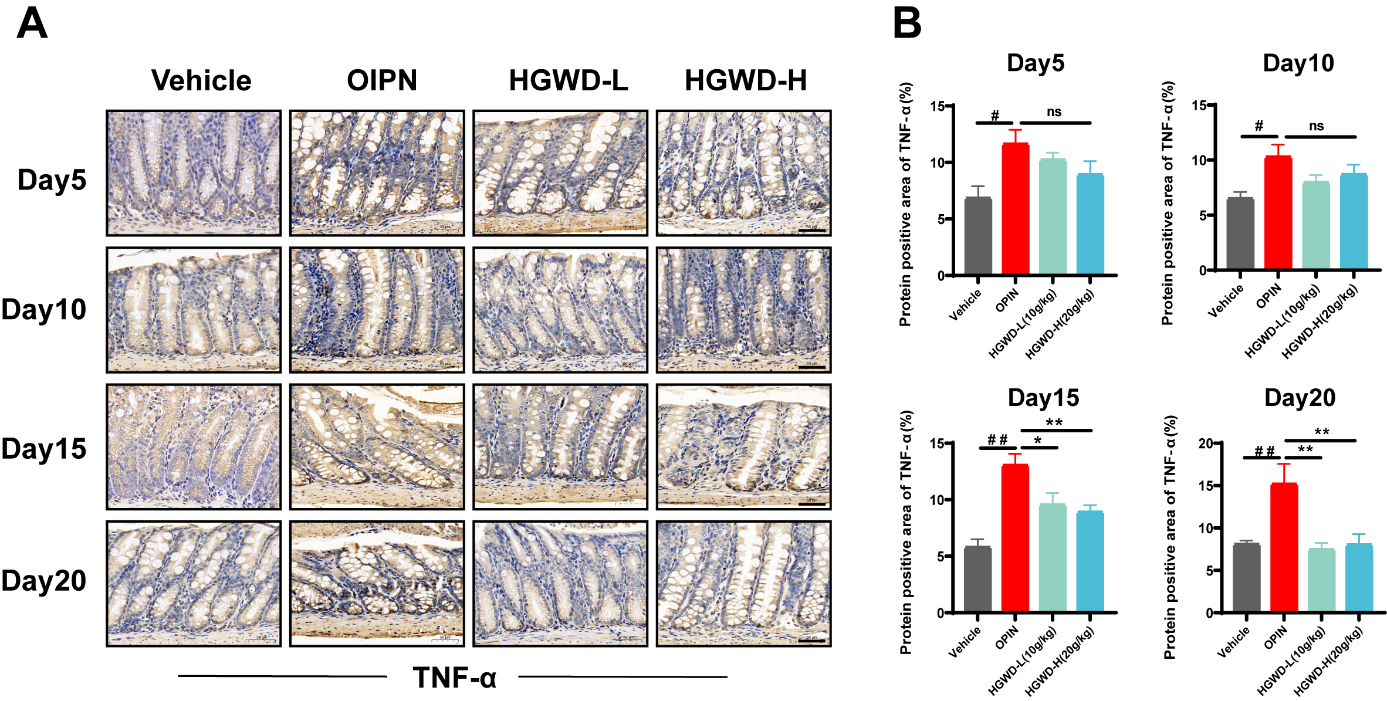


Figure S8. Effect of HGWD treatment on the expression level of TNF-α in the colon at different time periods (200×). (A) Colon TNF-α immunohistochemistry; (B) The expression level of TNF-α in the colon. # P<0.05, ## P<0.01 (compared with Vehicle group); * P<0.05 ** P<0.01 (compared with OIPN group). Abbreviations: HGWD-L, Huangqi Guizhi Wuwu Decoction low-dose; HGWD-H, Huangqi Guizhi Wuwu Decoction high-dose; OIPN, oxaliplatin-induced peripheral neurotoxicity; ns, not significant.
