# Supplementary material for: Huangqi Guizhi Wuwu decoction alleviates oxaliplatin-induced peripheral neuropathy via the gut-peripheral nerve axis
Source: Chin Med. 2023 Sep 7;18:114. doi: 10.1186/s13020-023-00826-5 (PMC10485938; doi:10.1186/s13020-023-00826-5)
Supplement: Supplementary file 2 — Additional file 2. Figure S9. Representative base peak intensity (BPI) chromatograms of HGWD analyzed by LC-MS in positive (A) and negative (B) mode. Table S2. Components of HGWD identified by LC-MS. Table S3. Calibration curves, limit of detection (LOD), limit of quantification (LOQ), accuracy, recovery, and contents of six analytes in HGWD. Table Sa4. Active compounds and their corresponding parameters in the HGWD formula. [file 13020_2023_826_MOESM2_ESM.docx]

**Additional file 2: Materials**


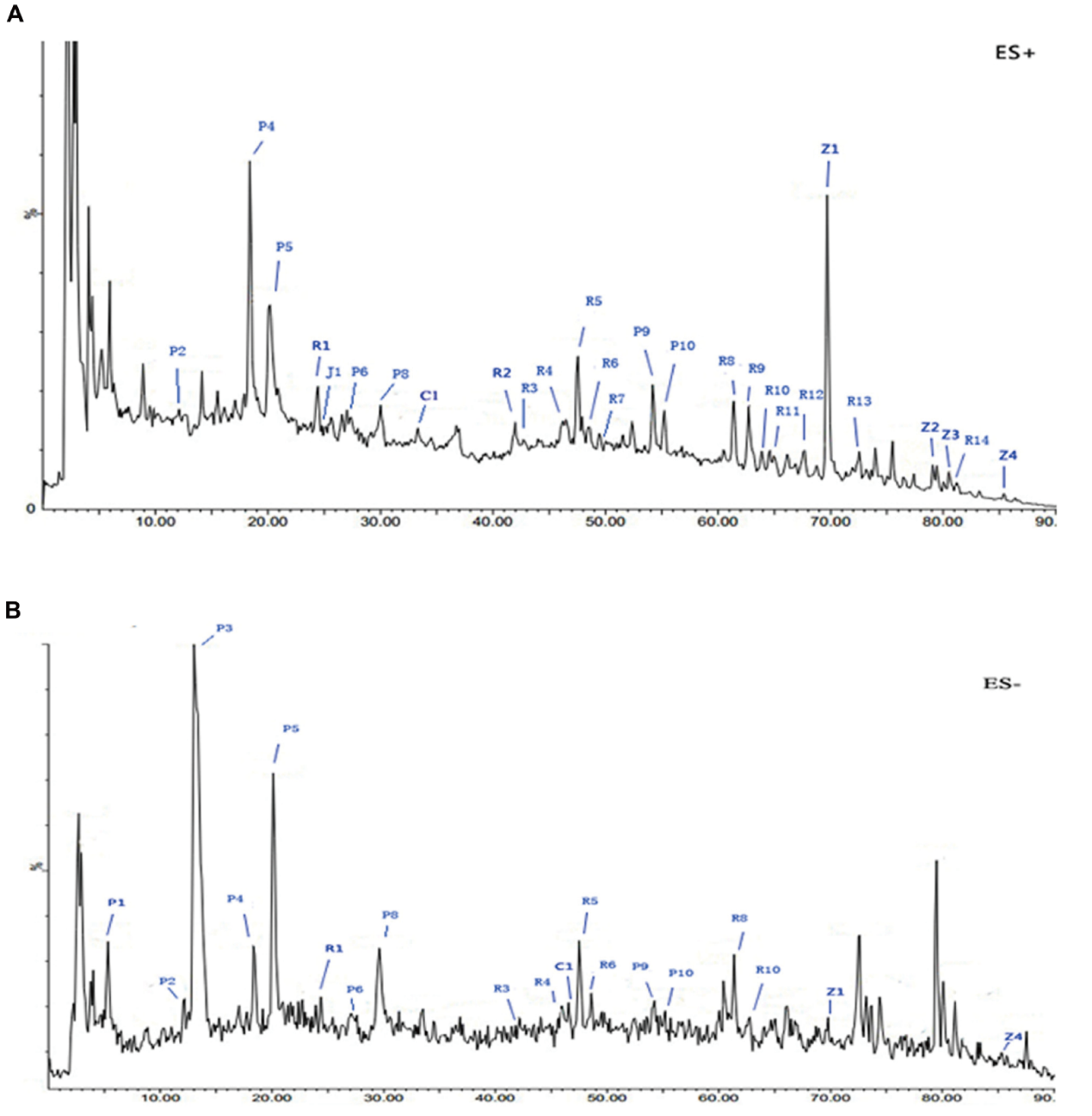


Figure S9. Representative base peak intensity (BPI) chromatograms of HGWD analyzed by LC-MS in positive (A) and negative (B) mode[10].

Table S2. Components of HGWD identiﬁed by LC-MS[10].

| **No.** | **Peak** | **t_R (min)_** | **Compound** | **Molecular weight** | **Formula** | ***m/z* (ES+)** | ***m/z* (ES-)** | **Herb** |
| --- | --- | --- | --- | --- | --- | --- | --- | --- |
| 1 | P1 | 5.27 | Gallic acid | 170.1 | C_7_H_6_O_5_ | n | 169 | Paeoniae Radix Alba |
| 2 | P2 | 11.95 | Oxypaeoniflora | 496.5 | C_23_H_28_O_12_ | 519 | 495;555 | Paeoniae Radix Alba |
| 3 | P3 | 13.01 | Paeoniflorin sulfonate | 544.1 | C_23_H_28_0_13_S | n | 543 | Paeoniae Radix Alba |
| 4 | P4 | 18.37 | Albiflorin | 480.5 | C_23_H_28_O_11_ | 481;503 | 479;539 | Paeoniae Radix Alba |
| 5 | P5 | 20.10 | Paeoniflorin | 480.5 | C_23_H_28_O_11_ | 481;503 | 479;539 | Paeoniae Radix Alba |
| 6 | R1 | 24.35 | calycosin-7-O-β-D-glucoside | 446.4 | C_22_H2_2_O_10_ | 285;447 | 283 | Paeoniae Radix Alba |
| 7 | J1 | 25.12 | Rutin | 610.5 | C_27_H_30_O_16_ | 611 | 609 | Astragali Radix |
| 8 | P6 | 27.35 | Galloylpaeoniflorin | 632.6 | C_30_H_32_O_15_ | 655 | 631 | Jujubae Fructus |
| 9 | P7 | 27.77 | Benzoic acid | 122.1 | C_7_H_6_O_2_ | n | 121 | Paeoniae Radix Alba |
| 10 | P8 | 29.58 | Pentagalloylglucose | 940.7 | C_41_H_32_O_26_ | n | 939 | Paeoniae Radix Alba |
| 11 | C1 | 33.32 | Coumarin | 146.2 | C_9_H_6_O_2_ | 147 | n | Cinnamomi Ramulus |
| 12 | R2 | 41.90 | Ononin | 430.4 | C_22_H_22_O_9_ | 269;431 | n | Astragali Radix |
| 13 | R3 | 42.61 | Calycosin-7-O-β-D-glc-6''-O-acetate | 488.1 | C_24_H_24_O_11_ | 489;511;285 | n | Astragali Radix |
| 14 | R4 | 46.15 | 3,9-dimethoxypterocarpane-10-O-β-D-glucopyranoside | 462.0 | C_23_H_26_O_10_ | 485;301 | n | Astragali Radix |
| 15 | C2 | 46.60 | Cinnamic acid | 148.2 | C_9_H_8_O_2_ | n | n | Cinnamomi Ramulus |
| 16 | R5 | 47.47 | Calycosin | 284.3 | C_16_H_12_O_5_ | 285 | 283 | Astragali Radix |
| 17 | R6 | 48.47 | 2’-hydroxy-3’,4’-dimethoxyisoflavane-7-O-β-D-glucopyranoside. | 464.0 | C_23_H_28_O_10_ | 487;303 | n | Astragali Radix |
| 18 | C3 | 49.38 | Cinnamaldehyde | 132.2 | C_9_H_8_O | n | n | Cinnamomi Ramulus |
| 19 | R7 | 49.48 | Formononetin-7-O-β-D-glc-6''-O-malonate | 516.1 | C_25_H_24_O_12_ | 517;269 | n | Astragali Radix |
| 20 | C4 | 52.27 | unknown |  |  | 161;106 | n | Cinnamomi Ramulus |
| 21 | P9 | 54.18 | Benzoylalbiflorin | 584.6 | C_30_H_32_O_12_ | 607 | 583 | Paeoniae Radix Alba |
| 22 | P10 | 55.12 | Benzoylpaeoniflorin | 584.6 | C_30_H_32_O_12_ | 607 | 583 | Paeoniae Radix Alba |
| 23 | C5 | 57.66 | unknown |  |  | 163; |  | Cinnamomi Ramulus |
| 24 | R8 | 60.50 | Unknown |  |  | 353;295 | 329 | Astragali Radix |
| 25 | R9 | 61.33 | Formononetin | 268.3 | C_16_H_12_O_4_ | 269 | 267 | Astragali Radix |
| 26 | R10 | 62.67 | 3’-hydroxy-9,10-dimethoxypterocarpane | 300.3 | C_17_H_16_O_5_ | 301 | n | Astragali Radix |
| 27 | R11 | 63.09 | Astragaloside IV | 784.9 | C_41_H_68_O_14_ | 807 | 843 | Astragali Radix |
| 28 | R12 | 64.55 | 7,2-Dihydroxy-3,4-dimethoxyisoflavan | 302.3 | C_17_H_18_O_5_ | 303 | n | Astragali Radix |
| 29 | R13 | 67.59 | Astragaloside II | 827.0 | C_43_H_70_O_15_ | 849 | 886 | Astragali Radix |
| 30 | C5 | 66.30 | unknown |  |  | n | 295;233;147 | Cinnamomi Ramulus |
| 31 | Z1 | 69.67 | 6-gingerol | 294.4 | C_17_H_26_O_4_ | 317;137 | 293 | Zingiberis Rhizoma Recens |
| 32 | R14 | 70.20 | Isoastragaloside II | 827.0 | C_43_H_70_O_15_ | 849 | n | Astragali Radix |
| 33 | Z2 | 79.07 | 8-gingerol | 322.4 | C_19_H_30_O_4_ | 137 | 321 | Zingiberis Rhizoma Recens |
| 34 | Z3 | 80.48 | 6-shogaol | 276.4 | C_17_H_24_O_3_ | 277 | n | Zingiberis Rhizoma Recens |
| 35 | R15 | 80.63 | Acetylastragaloside I | 911.0 | C_47_H_74_O_17_ | 934 | n | Astragali Radix |
| 36 | Z4 | 85.33 | 10-gingerol | 350.5 | C_21_H_34_O_4_ | n | 349 | Zingiberis Rhizoma Recens |

Table S3. Calibration curves, limit of detection (LOD), limit of quantification (LOQ), accuracy, recovery, and contents of six analytes in HGWD[10].


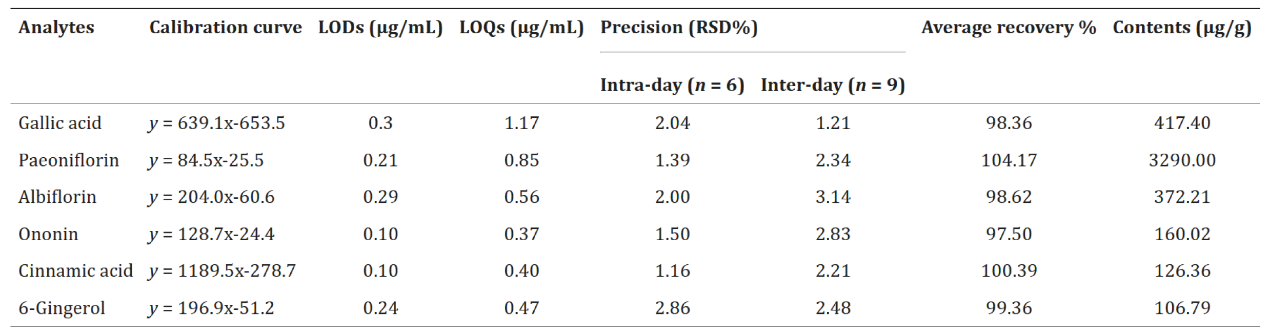


10. Cheng X, Huo J, Wang D, Cai X, Sun X, Lu W, Yang Y, Hu C, Wang X, Cao P. Herbal Medicine AC591 Prevents Oxaliplatin-Induced Peripheral Neuropathy in Animal Model and Cancer Patients. Front Pharmacol. 2017;8:344.

Table Sa4. Active compounds and their corresponding parameters in the HGWD formula[11].

| No | CAS | Compounds | Herb | Degree | Structure | |
| --- | --- | --- | --- | --- | --- | --- |
| 1 | 480-18-2 | Taxifolin | CR/ AR | 13 |  |  |
| 2 | 486-39-5 | (S)-Coclaurine | JF | 13 |  |  |
| 3 | 470-82-6 | 1,8-cineole | ZR | 10 |  |  |
| 4 | 99-50-3 | 3,4-dihydroxybenzoicacid | CR | 20 |  |  |
| 5 | N/A | 3,9-di-O-methylnissoln | AR | 11 |  |  |
| 6 | 77398-90-4 | 4-gingerol | ZR | 1 |  |  |
| 7 | 23513-14-6 | 6-gingerol | ZR | 21 |  |  |
| 8 | 555-66-8 | 6-shogaol | ZR | 17 |  |  |
| 9 | 2086-83-1 | Berberine | JF | 19 |  |  |
| 10 | 20575-57-9 | Calycosin | AR | 20 |  |  |
| 11 | 104-55-2 | Cinnamaldehyde | CR | 25 |  |  |
| 12 | N/A | Cinnamic acid | CR | 23 |  |  |
| 13 | N/A | Coumestrol | JF | 15 |  |  |
| 14 | 485-72-3 | Formononetin | AR | 38 |  |  |
| 15 | 130-86-9 | Fumarine | JF | 14 |  |  |
| 16 | 465-99-6 | Hederagenin | AR | 10 |  |  |
| 17 | 480-19-3 | Isorhamnetin | AR | 31 |  |  |
| 18 | 3301-49-3 | Jaranol | AR | 15 |  |  |
| 19 | 520-18-3 | Kaempferol | AR/ PRA | 45 |  |  |
| 20 | 32383-76-9 | Medicarpin | AR | 3 |  |  |
| 21 | 117-39-5 | Quercetin | AR/ JF | 69 |  |  |
| 22 | 92-61-5 | Scopoletin | JF | 15 |  |  |
| 23 | 2810-21-1 | Stepharine | JF | 11 |  |  |
| 24 | 16562-13-3 | Stepholidine | JF | 3 |  |  |
| 25 | 83-48-7 | Stigmasterol | AR/ZR/ JF | 17 |  |  |
| 26 | 122-48-5 | Zingerone | ZR | 11 |  |  |
| 27 | 83-46-5 | Beta-sitosterol | CR/ ZR/ PRA/ AR/ JF | 22 |  |  |
| 28 | 23180-57-6 | Paeoniflorin | AR/PRA | 14 |  |  |
| 29 | 472-15-1 | Betulic acid | PRA/ AR/ JF | 2 |  |  |
| 30 | 73536-69-3 | Bifendate | AR | 6 |  |  |

11. Lv Z, Shen J, Gao X, Ruan Y, Ling J, Sun R, Dai J, Fan H, Cheng X, Cao P. Herbal formula Huangqi Guizhi Wuwu decoction attenuates paclitaxel-related neurotoxicity via inhibition of inflammation and oxidative stress. Chin Med. 2021;16(1):76.
